# Model-Informed Radiopharmaceutical Therapy Optimization: A Study on the Impact of PBPK Model Parameters on Physical, Biological, and Statistical Measures in ^177^Lu-PSMA Therapy

**DOI:** 10.3390/cancers16183120

**Published:** 2024-09-10

**Authors:** Hamid Abdollahi, Ali Fele-Paranj, Arman Rahmim

**Affiliations:** 1 Department of Radiology, University of British Columbia, Vancouver, BC V5Z 1M9, Canada; 2 Department of Integrative Oncology, BC Cancer Research Institute, Vancouver, BC V5Z 1L3, Canada; alifele@student.ubc.ca; 3 Department of Mathematics, University of British Columbia, Vancouver, BC V5Z 1M9, Canada; 4 Department of Physics & Astronomy, University of British Columbia, Vancouver, BC V5Z 1M9, Canada

**Keywords:** radiopharmaceutical therapy, metastatic castration-resistant prostate cancer, physiologically based pharmacokinetic model, radiobiological response, biologically effective dose

## Abstract

**Simple Summary:**

This study investigates the influence of various pharmacokinetic parameters on ^177^Lu-PSMA therapies via physical, biological, and statistical measures. Employing a clinically validated physiologically based pharmacokinetic (PBPK) model, realistic time–activity curves (TACs) in tumors, salivary glands, and kidneys are generated, allowing for the calculation of metrics such as AUC, dose, BED, and fBED. The results demonstrate the significant impact of multiple parameters on the measured outcomes. In addition to the great impact of administered ligand amount, tumor volume, and receptor density, other pharmacokinetic parameters such as rates of radiopharmaceutical association, internalization, and release were identified as key influencers. Notably, alterations in the parameters induced distinct modifications in TAC features, affecting radiobiological, pharmacokinetic, and statistical aspects. These insights contribute to advancing personalized treatment regimens by understanding the key contributions of various parameters towards improving therapeutic efficacy while minimizing radiation-related toxicities.

**Abstract:**

***Purpose:*** To investigate the impact of physiologically based pharmacokinetic (PBPK) parameters on physical, biological, and statistical measures in lutetium-177-labeled radiopharmaceutical therapies (RPTs) targeting the prostate-specific membrane antigen (PSMA). ***Methods:*** Using a clinically validated PBPK model, realistic time–activity curves (TACs) for tumors, salivary glands, and kidneys were generated based on various model parameters. These TACs were used to calculate the area-under-the-TAC (AUC), dose, biologically effective dose (BED), and figure-of-merit BED (fBED). The effects of these parameters on radiobiological, pharmacokinetic, time, and statistical features were assessed. ***Results:*** Manipulating PBPK parameters significantly influenced AUC, dose, BED, and fBED outcomes across four different BED models. Higher association rates increased AUC, dose, and BED values for tumors, with minimal impact on non-target organs. Increased internalization rates reduced AUC and dose for tumors and kidneys. Higher serum protein-binding rates decreased AUC and dose for all tissues. Elevated tumor receptor density and ligand amounts enhanced uptake and effectiveness in tumors. Larger tumor volumes required dosimetry adjustments to maintain efficacy. Setting the tumor release rate to zero intensified the impact of association and internalization rates, enhancing tumor targeting while minimizing the effects on salivary glands and kidneys. ***Conclusions:*** Optimizing PBPK parameters can enhance the efficacy of lutetium-177-labeled RPTs targeting PSMA, providing insights for personalized and effective treatment regimens to minimize toxicity and improve therapeutic outcomes.

## 1. Introduction

Prostate cancer is a prevalent malignancy among men worldwide, with metastatic castration-resistant prostate cancer (mCRPC) being an advanced and aggressive form of the disease [1]. Traditional treatment options for mCRPC are often limited in their effectiveness, prompting the exploration of novel therapeutic approaches [2]. Radiopharmaceutical therapies (RPTs) utilizing targeted radioactive agents have emerged as a promising modality for the management of mCRPC, offering potential for both localized tumor control and systemic disease targeting [3]. In recent years, tremendous excitement and development has taken place involving lutetium-177-labeled pharmaceuticals targeting prostate-specific membrane antigens (^177^Lu-PSMA RPTs), having demonstrated promising outcomes in terms of tumor response and overall survival [4,5]. However, the optimization and personalization of treatment protocols remains an ongoing challenge [6].

In recent years, model-informed drug discovery and development (MIDDD) has gained significant traction as a robust approach for optimizing therapeutic interventions [7]. Leveraging computational models, such as physiologically based pharmacokinetic (PBPK) models, has become a pivotal strategy endorsed by regulatory bodies such as the FDA for rational drug design, dosing regimen selection, and treatment optimization [8,9]. PBPK models integrate physiological and pharmacokinetic data with mathematical modeling techniques to predict the distribution and fate of a drug within the body [10]. In the context of RPTs, PBPK models capture critical factors such as radiopharmaceutical uptake, distribution, and elimination to estimate the radiation dose delivered to the tumor and healthy tissues [11,12]. These models serve as a versatile framework for optimizing and personalizing RPTs, integrating seamlessly with the concept of digital twinning for individual cancer patients [11].

In our recent efforts, the concept of theranostic digital twins (TDTs) for optimization of RPTs has also been raised [13,14,15]. In this approach, PBPK modelling can act as the main engine of TDTs, which enables us to create dynamic and patient-specific replicas of RPT processes. By capturing system- and drug-specific data, the TDTs will endeavor to predict the radiopharmaceutical injection profile (e.g., activity, timing, and fractionation), dose, and therapy outcomes. However, the accuracy and predictive capabilities of PBPK models heavily rely on the selection and estimation of model parameters. These parameters encompass various pharmaceutical, physiological, anatomical, and patient-specific characteristics that influence the simulation of final response in terms of physical and biological dose [16]. Understanding the impact of these PBPK parameters is crucial for refining treatment strategies and improving patient outcomes.

On the other hand, the field of radiobiological modeling in RPTs represents a burgeoning area of research distinct from conventional external beam radiotherapies (EBRTs) [17]. Unlike EBRTs, where dose calculations are well established, determining the RPTs’ dose involves an intricate time–activity curve (TAC), which is intricately tied to pharmacokinetic parameters [16]. Modeling RPT responses in terms of biological dose is not as clearly defined as in EBRTs and warrants refinement, incorporating time-specific factors like cellular proliferation and repair mechanisms [17]. Moreover, the unique exponential increase and subsequent decrease in dose delivery rates in RPTs introduce novel radiobiological phenomena, such as adaptive responses, necessitating their inclusion in modeling efforts [18]. Additionally, the shape of the time–activity curve can serve as a snapshot of dose delivery characteristics in each tissue, offering a potentially predictive indicator for RPT outcomes. Exploring this facet further holds promise in the realm of personalized RPTs, highlighting the need for ongoing investigation and refinement in this rapidly advancing field.

In this study, we investigate the impact of PBPK parameters on physical, biological, and statistical measures in ^177^Lu-PSMA therapy for mCRPC. Through a model-informed design, we analyze different PBPK parameters to elucidate their influence on the radiation dose delivered to the tumor and PSMA-positive healthy tissues, including kidney and salivary glands. Furthermore, in this comprehensive analysis, we evaluate the biologically effective dose (BED). Subsequently, we explore how PBPK parameters influence the shape of the TACs concerning pharmacokinetic, radiobiological, and statistical features. This study represents a step towards optimizing RPTs for mCRPC, ultimately aiming to improve treatment outcomes and enhance the quality of life for patients with metastatic castration-resistant prostate cancer.

## 2. Material and Methods

### 2.1. PBPK Model Development

A PBPK model was developed to simulate the distribution and behavior of the ^177^Lu-PSMA within the body. PBPK models leverage ordinary differential equations (ODEs) to simulate the dynamic behavior of pharmaceuticals within the body post-injection [19]. Our model intricately incorporates major organs, treating their structures as discrete compartments. The intercompartmental flow of species is precisely governed by ODEs, enabling a detailed and dynamic depiction of the complex interactions and distribution kinetics within the physiological system. The model is made publicly available at: https://github.com/alifele/Computational-Physics/tree/main/PSMA-PBPK-Model-Matlab (accessed on 1 August 2024). The structure, compartments, and sub-compartments are detailed in Supplementary Figure S1, and more details about the model can also be found in [20].

Our developed PBPK model was validated by comparing the simulated results with available clinical data from previous studies [20,21]. The model’s ability to accurately predict the TACs of the ^177^Lu-PSMA in various organs of interest, such as tumors, salivary glands, and kidneys, was assessed. Good agreement between the simulated and observed data provided confidence in the model’s accuracy and suitability for further analysis. In line with previous studies [22], our methodology involves presenting results for a single cycle and assumes a typical injection scenario. Specifically, we administer 5.4 GBq Lu-177-PSMA composed of 7.5 nmol of hot and 91 nmol of cold radiopharmaceutical.

### 2.2. Testing Model Parameters

Using the validated PBPK model, a range of theoretical values for various tumor pharmacokinetic/physiological and physical parameters associated with ^177^Lu-PSMA therapy were considered. These parameters included association rate, internalization rate, serum protein-binding rate, release rate, receptor density, ligand amount, and tumor volume. These parameters are detailed in Table 1. The default values for these parameters, as obtained from the key reference Kletting et al. [22], are shown in Supplementary Table S1. In brief, these values are as follows: association rate (0.046 L/nmol/min), internalization rate (0.001 L/min), serum protein-binding rate (0.00047 L/min), release rate (0.00024 L/min for tumors, 0.00037 L/min for kidneys and salivary glands), ligand amount (7.5 nmol), receptor density (500 nmol/L for tumors, 31 nmol/L for kidneys, 41 nmol/L for salivary glands), and tissue volume (0.01 L for tumors, 0.311 L for kidneys, 0.021 L for salivary glands). Subsequently, for these ranges of parameters, 728 TACs were generated. We should mention that when these parameters are altered, default parameters are utilized. For instance, the default tumor size is set at 0.01 L with a receptor density of 500 nmol/L. It is important to acknowledge that while tumors typically exhibit higher receptor densities compared to normal organs, there may be instances where theoretical assumptions suggest similarities between them. This theoretical assumption may occur during certain therapy cycles, particularly when tumors undergo substantial shrinkage, leading to changes in factors such as volume and receptor densities. Traditionally, tumor receptor density is expected to be higher than that of OARs, which is a cornerstone of effective therapy. Additional details on the parameters can be found in the Supplementary Materials [16,22,23,24,25].

### 2.3. Area under the TAC (AUC) and Dose

PBPK-simulated time–ligand concentration curves (TLCC) were used for the calculation of the area under the curve (AUC) and dose for tumors, salivary glands, and kidneys. In our model, TLCC represents the results of the simulation and is conceptually similar to TACs. Instead of displaying activity, TLCC shows the amount of the radiopharmaceutical in each organ, which can easily be converted to TAC. AUC was determined by quantifying the accumulated amount of the radiopharmaceutical ligand in nanomoles × minute using the following equation:

(1) $$AUC=\int_{0}^{{\;t}_{n}}TLCC\;\left(t\right)dt=\int_{0}^{{\;t}_{n}}\;N_{i}(t)dt$$

where $N_{i}$ is the amount of radiopharmaceutical ligands in nanomole. In this study, $t_{n}$ was assigned as 30,000 min.

Dose (*D*) was calculated through the following equations:

(2) $${\overset{•}{D}}_{i}(t)=N_{i}(t)\cdot \lambda \cdot S_{i\leftarrow i}$$

(3) $$D=\int_{0}^{{\;t}_{n}}\overset{•}{D_{i}}(t)dt$$

${\overset{•}{D}}_{i}\left(t\right)\;is\;dose\;rate,while\;\lambda \;and\;S_{i\leftarrow i}$ are physical decay constant and specific absorbed fraction, respectively. These values are shown in Supplementary Table S2.

### 2.4. Biologically Effective Dose (BED)

The numerous TACs obtained from the PBPK model were utilized to calculate the BEDs for the tumors, salivary glands, and kidneys. BED represents the radiation dose delivered to the tissues, taking into account factors such as the type of radiation delivery (fractionation or here as dose rate) and tissue-specific (e.g., radiosensitivity and repair). Based on ICRU report 96 [26], BED is defined as “the absorbed dose that is required to cause a given biological effect if the absorbed dose is delivered in infinitely small doses per fraction or, equivalently, at very low absorbed dose rates such as in low-dose-rate brachytherapy”. In this study, BED calculations were performed using four different radiobiological models. BED can be obtained using a linear quadratic (LQ) model expressed as $S=e^{-D(\alpha +\beta GD)}$, where α and β describe cellular radiosensitivity, *D* is the dose, and *G* is the dose protraction (Lea–Catcheside) factor [27] accounting for the impact of dose rate on the biological effect of radiation. Then, BED can be simply calculated as BED=D (1+GDαβ). In this study, we calculated *G* using four different formalisms, as elaborated below.

#### 2.4.1. G Described by Lea–Catcheside

The Lea–Catcheside G function, *G*(*T*), is a mathematical model designed to quantify the modifying effect of sub-lethal damage repair in response to variations in dose rate over time during radiation therapy. Originally developed within the context of the LQ model to describe the kinetics of chromosome breakage and repair, the *G* function provides a measure of the biological effectiveness of radiation when delivered in a fractionated or protracted manner. It captures the dynamic interplay between dose rate and the rate of repair, allowing for a more accurate prediction of radiation-induced damage under varying dose delivery conditions. It is given by:

(4) $$G(T)=\frac{2}{D^{2}}\int_{0}^{T}\dot{D}(t)dt\int_{0}^{t}\dot{D}\;(w)\;\mathrm{dw}\;e^{-µ(t-w)}dw$$

where *D* is dose, $\dot{D}$ is dose rate, and *μ* is the repair rate constant, assuming exponential repair, which indicates that the probability of a repair event decreases exponentially over time. The second integral represents the interval between breaks; *w* is the time of the first break, *t* is the time of the second break, and *T* is the irradiation duration, which we take as infinity in RPTs. This is the most general formulate for *G* [27], while formulas that follow are based on stronger assumptions.

#### 2.4.2. G as Simplified Version of Lea–Catcheside

For a tissue region where the absorbed dose rate decreases mono-exponentially with time, characterized by a rate constant, *λ*, the Lea–Catcheside *G* function simplifies to:

(5) $$G=\frac{\lambda }{\lambda +\mu }$$

where λ is effective decay and *μ* is the repair rate constant. This formulation can be arrived at by exponentially decreasing TACs [26].

#### 2.4.3. G Described by Kalogianni et al. [28]

Based on a study by Kalogianni et al. [28], for a biphasic radiopharmaceutical uptake, the G-factor in this study was determined to be:

(6) $$G=\frac{\lambda_{e}\lambda_{eu}{(\lambda }_{r}-\lambda_{e}-\lambda_{eu})}{{(\lambda }_{e}+\lambda_{eu}){(\lambda }_{r}-\lambda_{e}){(\lambda }_{r}-\lambda_{eu})}+\frac{2{\lambda_{e}}^{2}{\lambda_{eu}}^{2}}{\left({\lambda_{r}}^{2}-{\lambda_{e}}^{2})({\lambda_{r}}^{2}-{\lambda_{eu}}^{2}\right)}$$

where $\lambda_{e}\;$ is the effective clearance constant, ${\;\lambda }_{eu}$ is the effective uptake constant, and $\lambda_{r}$ is the repair coefficient (*μ*). This formulation assumes TACs that have both uptake and clearance rates [28].

#### 2.4.4. G Described by Howell et al. [29]

This G, calculated through Equation (7), provides a detailed formulation for radiopharmaceuticals in RPTs, considering incomplete decay and complex dose rate kinetics. It incorporates the uptake and clearance half-times of the radiopharmaceuticals in tissues, enabling a more accurate assessment of therapeutic effectiveness over time. This equation integrates various biological and physical decay parameters to evaluate the performance of different radiopharmaceuticals in treatment scenario.

(7) $$\begin{array}{l} G(t,T_{i}{\neq 0,T}_{d}\neq 0,T_{i}>T_{d})=\\ \;\;\;G\left(t;\;T_{i}\;\neq \;0,\;T_{d}\;\neq \;0,\;T_{d}\;>\;T_{i}\right)\;\\ \;\;\;\;\;\;\;\;\;\;\;\;=\;\frac{2r_{0}}{D\left(t\right)}\;\left(\;\frac{1}{\lambda_{d}}\;\left(1\;-\;e^{-\lambda_{d}\;t}\right)\;-\;\frac{1}{\lambda_{i}}\;\left(1\;-\;e^{-\lambda_{i}\;t}\right)\;\right)\;\\ \;\;\;\;\;\;\;\;\;\;\;\;\times \;\{\;\left(\;\frac{1}{\left(\lambda_{d}\;-\;\mu \right)}\;-\;\frac{1}{\left(\lambda_{i}\;-\;\mu \right)}\;\right)\\ \;\;\;\;\;\;\;\;\;\;\;\;\cdot \;\left(\;\frac{1}{\left(\mu \;+\;\lambda_{d}\right)}\;\left(1\;-\;e^{-\left(\mu \;+\;\lambda_{d}\right)\;t}\right)\;-\;\frac{1}{\left(\mu \;+\;\lambda_{i}\right)}\;\left(1\;-\;e^{-\left(\mu \;+\;\lambda_{i}\right)\;t}\right)\;\right)\\ \;\;\;\;\;\;\;\;\;\;\;\;+\;\left(\;\frac{1}{\left(\lambda_{d}\;-\;\mu \right)}\;+\;\frac{1}{\left(\lambda_{i}\;-\;\mu \right)}\;\right)\;\cdot \;\left(\;\frac{1}{\left(\lambda_{d}\;+\;\lambda_{i}\right)}\;\left(1\;-\;e^{-\left(\lambda_{i}\;+\;\lambda_{d}\right)\;t}\right)\;\right)\;\\ \;\;\;\;\;\;\;\;\;\;\;\;+\;\frac{1}{2\;\lambda_{d}\;\left(\lambda_{d}\;-\;\mu \right)}\;\left(1\;-\;e^{-2\lambda_{d}\;t}\right)\;-\;\frac{1}{2\;\lambda_{i}\;\left(\lambda_{d}\;-\;\mu \right)}\;\left(1\;-\;e^{-2\lambda_{i}\;t}\right)\;\} \end{array}$$

where *D* is dose, $r_{0}$ is initial dose rate, *µ* is the rate constant for exponential sublethal damage (SLD) repair with first order kinetics, *λ_d_* (*T_d_* = ln (2)/*λ_d_*) and *λ_i_* (*T_i_* = ln (2)/*λ_i_*) are the rate constants for exponentially decreasing and increasing dose rates, respectively, and *t* is the time over which irradiation takes place. By considering *t*→∞ (i.e., radioactivity stays in the body), Equation (8) simplifies to:

(8) $$\begin{array}{l} G\left(\infty ;T_{i}\neq 0,T_{d}\neq 0,T_{d}>T_{i},\;\right)=\frac{2r_{0}}{D\left(\infty \right)}\left(\frac{1}{\lambda_{d}}-\frac{1}{\lambda_{i}}\right)\times \{\left(\frac{1}{\left(\lambda_{d}-\mu \right)}-\frac{1}{\left(\lambda_{i}-\mu \right)}\right)\cdot \left(\frac{1}{\left(\mu +\lambda_{d}\right)}-\frac{1}{\left(\mu +\lambda_{i}\right)}\right)+\\ \left(\frac{1}{\left(\lambda_{d}-\mu \right)}+\frac{1}{\left(\lambda_{i}-\mu \right)}\right)\cdot \frac{1}{\left(\lambda_{d}+\lambda_{i}\right)}+\frac{1}{2\lambda_{d}\left(\lambda_{d}-\mu \right)}-\frac{1}{2\lambda_{i}\left(\lambda_{d}-\mu \right)}\} \end{array}$$

The values for *µ* are shown in Supplementary Table S2. $r_{0\;}$ Log curves of dose rate time–curves were generated, and back-extrapolation was used to measure $r_{0\;}$, as shown in Supplementary Figure S3.

Equation (7), utilized by Howell et al. [29] and Solanki et al. [18], evaluates the shape of the TAC consisting of increasing and decreasing aspects, as shown in Supplementary Figure S2. Typically, dose-rate patterns exhibit a common trend where they initially show an exponential rise until reaching a peak value, after which they exhibit an exponential decline. The dose rate increase half-time (*T_i_*) refers to the approximate time it takes for the dose rate to reach half of its maximum value. On the other hand, the dose rate decrease half-time (*T_d_*) represents the approximate time it takes for the dose rate to decrease to half of its maximum value (Supplementary Figure S2).

### 2.5. Figure-of-Merit Biological Effective Dose (fBED)

To calculate the effect of pharmacokinetic parameters on the overall treatment plans in terms of both effective tumor and healthy tissue doses, we calculated the figure-of-merit biological effective dose (*fBED*) using the following equation:

(9) $$fBED=1/\left[1+\frac{\sum_{i=1}^{n}\;{BED}_{OAR}^{i}}{\sum_{j=1}^{m}{BED}_{Tumor}^{j}}\right]$$

where *BED_OAR_* is the BED of the organs at risk, including the salivary glands and kidneys. In the case of tumors, just a single tumor was studied.

### 2.6. Sensitivity Analysis

A sensitivity analysis was conducted to assess the impact of changing individual pharmacokinetic parameters on the calculated AUC, dose, BED, and fBED values for tumors, salivary glands, and kidneys. The net effect of those parameters on treatment plans was measured as fBED.

### 2.7. The Effect of Offsetting Tumor Release Rate to Zero

Since radiopharmaceuticals are administered intravenously, they have the potential to bind to serum proteins, such as albumin, once introduced into the bloodstream [30]. Furthermore, the rate at which radiopharmaceuticals are released from the tumor can significantly influence the loss of therapeutic dosage [22]. As such, these factors have a significant impact on therapeutic applications. To thoroughly assess the role of the tumor release rate, we systematically tested key parameters, such as the association rate, internalization rate, tumor receptor density, tumor volume, and ligand amount, on AUC, dose, BED, and fBED when the tumor release rates were set to zero.

### 2.8. Time–Activity Features

We carried out an extraction of these six features from the TACs. The inclusion of two pharmacokinetic parameters, Cmax and time to peak, allowed us to analyze the peak concentration and the timing of its occurrence. Additionally, two radiobiological parameters, *Ti* and *Td*, offered insights into early and late decay phases in TACs (they are depicted in Supplementary Figure S2). Complementing these, two statistical measures, skewness and entropy, provided quantitative assessments of asymmetry and randomness within the TAC. Our elaborate feature extraction process aims to enrich the quantification of TAC dynamics. For comprehensive details on each extracted feature and its implications, please refer to Table 2. Furthermore, the effect of these parameters on the extracted features were also plotted.

### 2.9. Relative Biological Effectiveness (RBE)

To assess how biological effectiveness can be changed based on varying parameters compared to the default values, we incorporated a detailed analysis of the relative biological effectiveness (RBE). RBE measures the potency of radiation to produce a specific biological effect, calculated as the ratio of the absorbed dose of a reference radiation type (typically, standard photon radiation) to the absorbed dose of the radiation under investigation required to achieve the same biological effect. For this study, we focused on four critical parameters: association rate, internalization rate, release rate, and serum protein-binding rate. We calculated RBE using the following equation:

(10) $$RBE=\frac{D_{Reference}}{D_{Investigated}}$$

where $D_{Reference}$ is the dose derived using the default parameters and $D_{Investigated}$ is the dose obtained when each parameter was individually varied. This approach provided insights into how alterations in these parameters influence the RBE while maintaining a constant RBE for ^177^Lu.

## 3. Results

### 3.1. The Effect of PBPK Modeling Parameters on AUC, Dose, BED, and fBED

**Figure 1:**
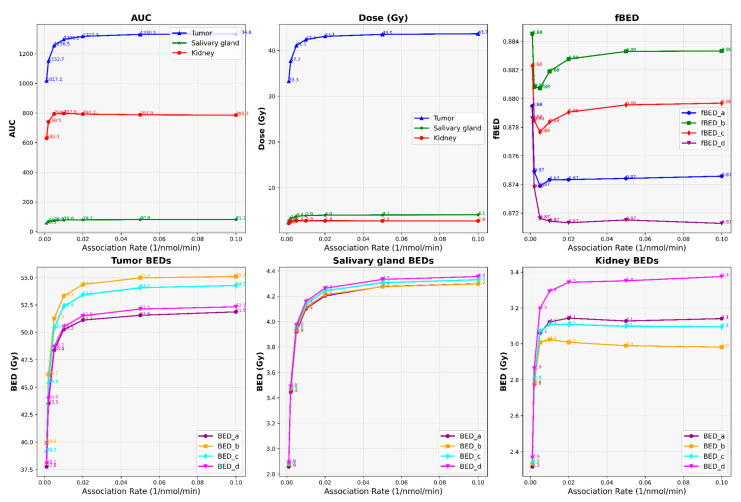
The impact of changes in association rate (L/nmol/min) on AUC, dose, BED, and fBED in tumors, salivary glands, and kidneys.

**Figure 2:**
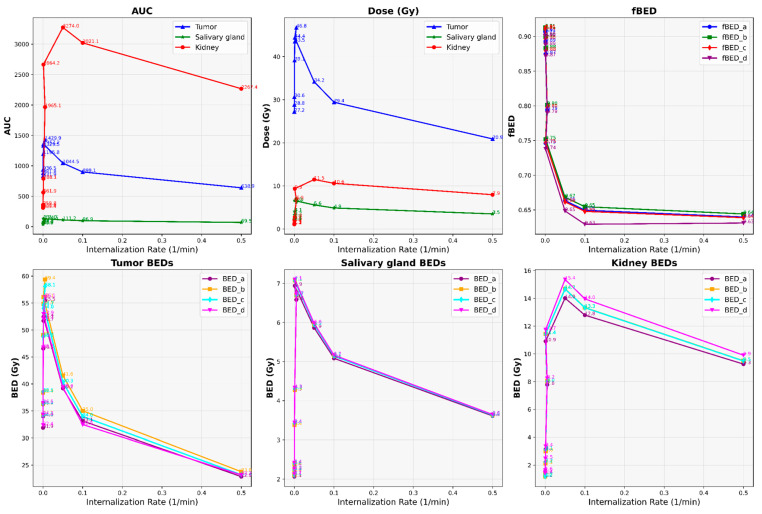
The impact of changes in internalization rate (L/min) on AUC, dose, BED, and fBED in tumors, salivary glands, and kidneys.

**Figure 3:**
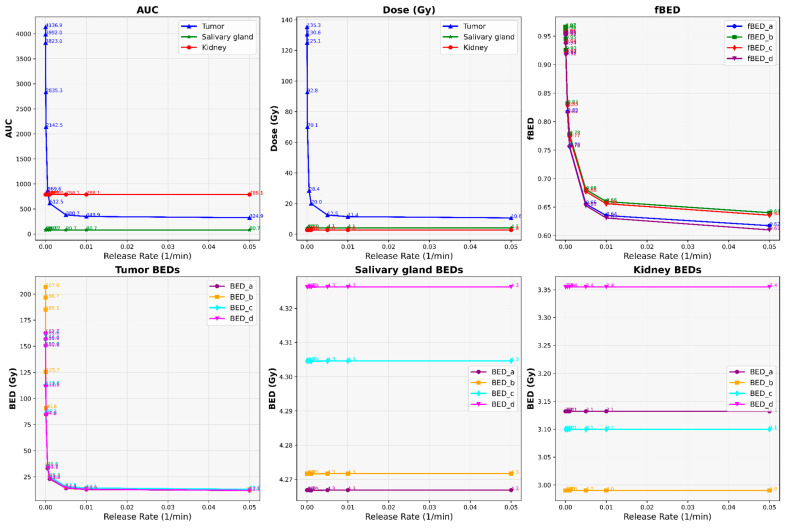
The impact of changes in tumor release rate (L/min) on AUC, dose, BED, and fBED in tumors, salivary glands, and kidneys.

**Figure 4:**
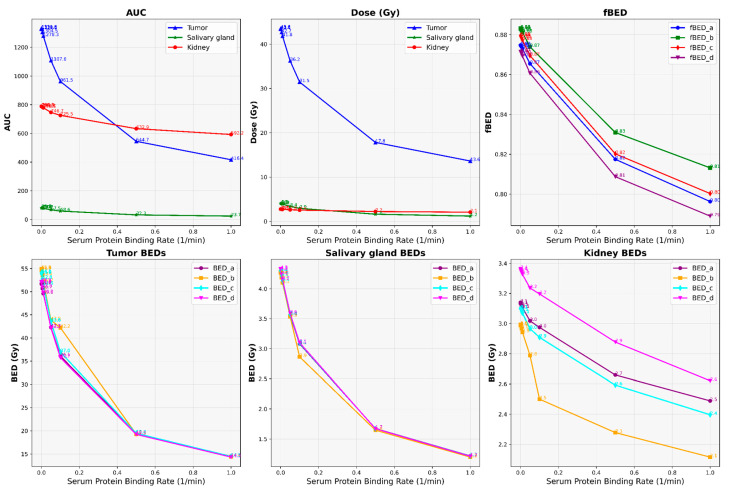
The impact of changes in serum protein-binding rate (L/min) on AUC, dose, BED, and fBED in tumors, salivary glands, and kidneys.

**Figure 5:**
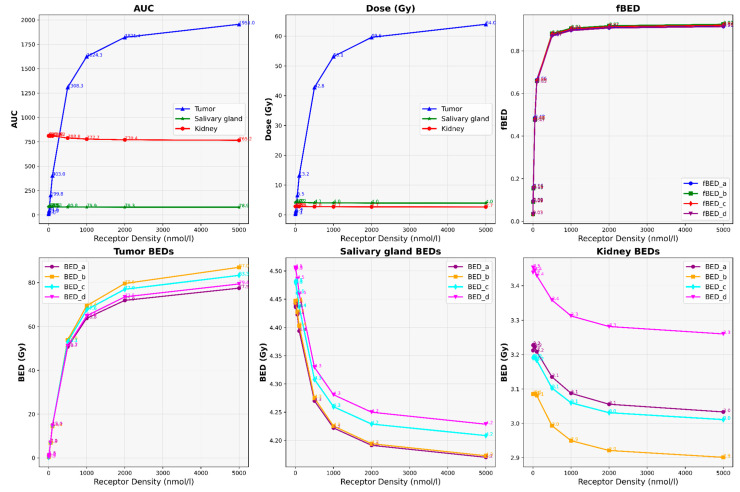
The impact of changes in tumor receptor density (nmol/L) on AUC, dose, BED, and fBED in tumors, salivary glands, and kidneys.

Our results depict the influence of manipulating chosen PBPK parameters on AUC, dose, BED, and fBED outcomes, as illustrated in Figure 1, Figure 2, Figure 3, Figure 4 and Figure 5, for association rate, internalization rate, serum release rate, serum protein-binding rate, and tumor volume, respectively. Supplementary Figures S4 and S5 show results for ligand amount and tumor volume respectively. In subsequent sections, we provide detailed explanations for the results of each parameter. To present the results for the four different BEDs/fBEDs more clearly, we use the following designations: (a) BED/fBED calculated using G from the simplified Lea–Catcheside method, (b) BED/fBED calculated using G from Kalogianni et al., (c) BED/fBED calculated using G from the original Lea–Catcheside method, and (d) BED/fBED calculated using G from Howell et al.

Figure 1 demonstrates the significant dependencies that radiopharmaceutical uptake and effectiveness have on the association rate (L/nmol/min). As the association rate increases, the AUC, dose, BED, and fBED values consistently rise across the tumors, salivary glands, and kidneys. For the tumors, the AUCs increase from 1017.23 at an association rate of 0.001 to 1334.60 at 0.1, while the dose similarly rises from 33.28 Gy to 43.66 Gy. BED values for the tumor show a parallel increase, with BED_L moving from 37.75 Gy to 51.87 Gy and similar upward trends observed in BED_G, BED_lambda, and BED_R0. The salivary gland also experiences a steady rise, with AUC increasing from 55.54 to 81.19 and dose from 2.79 Gy to 4.07 Gy. Correspondingly, BED values for the salivary glands increase, with BED_L ranging from 2.86 Gy to 4.30 Gy. The kidneys follow this trend as well, with their AUCs rising from 630.30 to 785.71, dose from 2.21 Gy to 2.75 Gy, and BED_L from 2.32 Gy to 3.14 Gy. The fBED values remain relatively stable across all of the organs, with only slight variations appearing as the association rate increases. Overall, the increasing association rates enhance the radiopharmaceutical uptake and dose delivery across all of the organs, with the most significant improvements observed in the tumors, while the salivary glands and kidneys experience more moderate gains.

Figure 2 illustrates how varying the internalization rate (L/min) influences the AUC, dose (Gy), BED, and fBED for tumors, salivary glands, and kidneys. As the internalization rate increases, the results for AUC, dose, BED, and fBED across the tumors, salivary glands, and kidneys exhibit distinct patterns. For the tumors, both AUC and dose initially rise, with AUC peaking at 1429.86 and dose at 46.78 Gy at an internalization rate of 0.005, before declining significantly to 638.86 and 20.90 Gy at the highest rate of 0.5. BED values for the tumors, calculated using different methods, follow a similar trend, with values peaking from around 55.45 Gy to 59.38 Gy and then decreasing to a range between 22.86 Gy and 23.17 Gy. In contrast, the kidney shows a more pronounced increase in both AUC and dose, peaking between 3274.00 and 11.47 Gy at an internalization rate of 0.05, before declining to between 2267.38 and 7.95 Gy at 0.5. BED values for the kidney exhibit a similar pattern, peaking around 14.69 Gy and then slightly decreasing. The salivary gland shows a moderate increase in AUC and dose, reaching between 131.00 and 6.57 Gy at 0.01 before declining to between 69.54 and 3.49 Gy at 0.5, with BED values peaking around 6.70 Gy. Across all organs, fBED values decrease consistently as the internalization rate increases, with tumor fBED values dropping from 0.9079 to 0.6398, indicating a general reduction in biological effectiveness at higher internalization rates. Overall, while moderate internalization rates optimize radiopharmaceutical delivery, higher rates diminish its effectiveness across all tissues.

The findings, as illustrated in Figure 3, demonstrate that augmenting the release rate results in a reduction in AUC, dose, and BED across all organs. As the release rate increases, there is a consistent decrease in the AUC, dose, BED, and fBED values for the tumors, while the salivary glands and kidneys remain largely unaffected. For the tumors, the AUCs drop from 4136.90 to 324.90, and the doses decrease from 135.34 Gy to 10.63 Gy as the release rate increases from 0.000001 to 0.05. Similarly, BED values for the tumors, across all calculation methods, show a significant decline, with BED_L decreasing from 162.67 Gy to 11.94 Gy, and comparable decreases observed for BED_G, BED_lambda, and BED_R0. In contrast, the AUC and dose for the salivary glands and kidney remains stable, with AUCs around 80.74 for the salivary glands and 788.06 for the kidneys, and the dose consistently at 4.05 Gy and 2.76 Gy, respectively. The BED values for these organs also show minimal variation, reflecting their relative insensitivity to changes in the release rate. The fBED values for the tumors decline across all methods, indicating reduced biological effectiveness as the release rate increases, with fBED_L dropping from 0.9565 to 0.6174. Overall, the increasing release rate significantly reduces the effectiveness of treatment for the tumors, while the salivary glands and kidneys are largely unaffected, suggesting a potential trade-off between tumor control and sparing non-target organs.

Figure 4 illustrates the impact of varying the serum protein-binding rate (L/min) on AUC, dose (Gy), BED, and fBED for tumors, salivary glands, and kidneys. As the serum protein-binding rate increases, the AUC, dose, BED, and fBED values consistently decrease across the tumors, salivary glands, and kidneys. For the tumors, AUC drops from 1331.59 at the lowest binding rate (0.0001) to 416.36 at the highest rate (1), while the dose decreases from 43.56 Gy to 13.62 Gy. Similarly, BED values for the tumors show a significant decline, with BED_L reducing from 51.78 Gy to 14.48 Gy, and similar decreases are observed in BED_G, BED_lambda, and BED_R0. The salivary glands also experience a decrease, with AUC falling from 80.86 to 23.65 and dose from 4.06 Gy to 1.19 Gy. Correspondingly, BED values for the salivary glands decrease, with BED_L dropping from 4.28 Gy to 1.22 Gy. The kidneys show a reduction in AUC from 788.46 to 592.23 and a dose decrease from 2.76 Gy to 2.08 Gy, with BED_L decreasing from 3.14 Gy to 2.49 Gy. The fBED values decrease across all organs, with the tumor’s fBED_L dropping from 0.8747 to 0.7963. Overall, increasing the serum protein-binding rate reduces the radiopharmaceutical uptake, dose delivery, and biological effectiveness across all organs, with the tumor showing the most significant decline in treatment efficacy.

Figure 5 demonstrates that as tumor receptor density increases, the AUC, dose, BED, and fBED values for the tumors show a significant upward trend, while the values for the salivary glands and kidneys remain relatively stable. The tumors’ AUCs rise sharply from 8.05 at the lowest receptor density (1) to 1955.04 at the highest density (5000), and the doses increase from 0.26 Gy to 63.96 Gy. The BED values for the tumor also increase substantially, with BED_L rising from 0.27 Gy to 77.47 Gy. In contrast, the AUC for the salivary gland remains around 83.86 at low receptor densities, slightly decreasing to 78.91 at the highest density. The dose to the salivary gland decreases modestly from 4.21 Gy to 3.96 Gy, with BED_L decreasing from 4.44 Gy to 4.17 Gy. The kidneys’ AUCs remain stable, decreasing slightly from 810.69 to 765.22, with the doses decreasing from 2.84 Gy to 2.68 Gy and BED_L decreasing from 3.21 Gy to 3.03 Gy. fBED values for the tumor increase steadily with receptor density, rising from 0.03 to 0.91, while fBED values for the salivary glands and kidneys remain relatively stable. Overall, increasing tumor receptor density significantly enhances radiopharmaceutical uptake and dose delivery to tumors, leading to substantial increases in BED and fBED, while the salivary glands and kidneys remain largely unaffected, indicating a favorable therapeutic index.

Supplementary Figure S4 illustrates the impact of increasing ligand amount (nmol) on AUC, dose (Gy), BED, and fBED in tumors, salivary glands, and kidneys. As the ligand amount increases, the AUC, dose, BED, and fBED values for the tumors exhibit a significant upward trend, with similar but less pronounced increases observed in the salivary glands and kidneys. For the tumors, AUC rises sharply from 177.71 at the lowest ligand amount (1) to 16,824.31 at the highest amount (100), while the dose increases from 5.81 Gy to 550.41 Gy. BED values for the tumors also increase substantially, with BED_L rising from 5.96 Gy to 1926.45 Gy, and similar trends are observed in BED_G, BED_lambda, and BED_R0. The salivary glands show a marked increase in AUC from 10.95 to 830.68, with the dose rising from 0.55 Gy to 41.67 Gy. Corresponding BED values for the salivary gland increase, with BED_L growing from 0.55 Gy to 65.72 Gy. The kidneys also experience an increase in AUC from 108.15 to 7628.41, with the dose rising from 0.38 Gy to 26.73 Gy. BED values for the kidneys follow a similar upward trend, with BED_L increasing from 0.39 Gy to 57.34 Gy. fBED values for all three organs increase steadily with the ligand amount, reflecting enhanced biological effectiveness. For the tumor, fBED_L rises from 0.86 to 0.94, and similar trends are observed in the salivary glands and kidneys. Overall, increasing the ligand amount significantly enhances radiopharmaceutical uptake, dose delivery, and biological effectiveness across all organs, with the tumor showing the most substantial improvements, indicating a strong positive correlation between ligand amount and treatment efficacy.

Supplementary Figure S5 shows the impact of changes in tumor volume (L) on AUC, dose (Gy), BED, and fBED for tumors, salivary glands, and kidneys. As the tumor volume increases, the AUC, dose, BED, and fBED values exhibit notable changes across the tumors, salivary glands, and kidneys. The AUC for the tumors escalates dramatically from 41.75 to 17,005.75 as the tumor volume increases from 0.001 to 1, indicating a substantial rise in radiopharmaceutical uptake in the tumor. In contrast, the AUC for the salivary glands and kidneys decreases, reflecting a reduced uptake in these organs with larger tumor volumes. The doses to the tumors decrease from 41.61 to 18.08, while doses to the salivary glands and kidneys also decline, with the kidney doses showing a more pronounced reduction. BED values for the tumors decrease across all calculation methods (L, G, lambda, and R0), with BED_L dropping from 70.99 to 19.33 and BED_R0 reducing from 60.12 to 20.08, indicating a reduction in the biological effectiveness of the treatment as the tumor volume grows. The fBED values for the tumors also show a slight decrease, with fBED_L decreasing from 0.90 to 0.83 and fBED_R0 from 0.88 to 0.83. These results suggest that as tumor volume increases, treatment effectiveness diminishes, necessitating adjustments in dosimetry to maintain optimal therapeutic outcomes.

### 3.2. Impact of PBPK Modeling Parameters on AUC, Dose, BED, and fBED by Considering a Zero Value of the Tumor Release Rate

**Figure 6:**
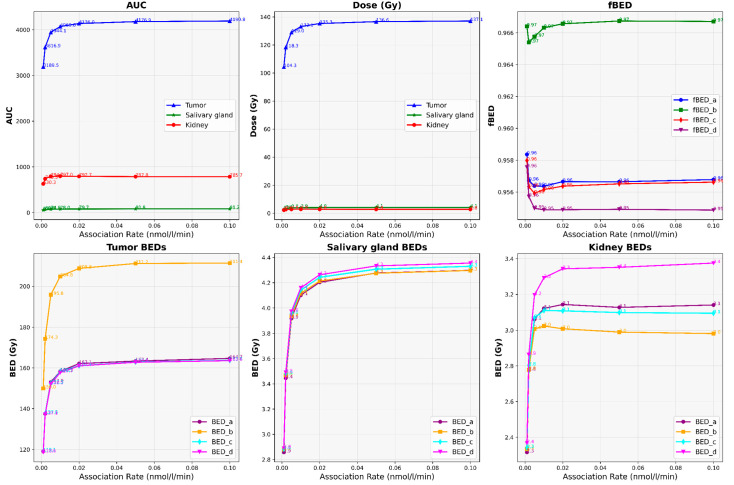
The impact of changes in association rate (nmol/L/min) on AUC, dose, BED, and fBED in tumors, salivary glands, and kidneys when tumor release rate is zero.

**Figure 7:**
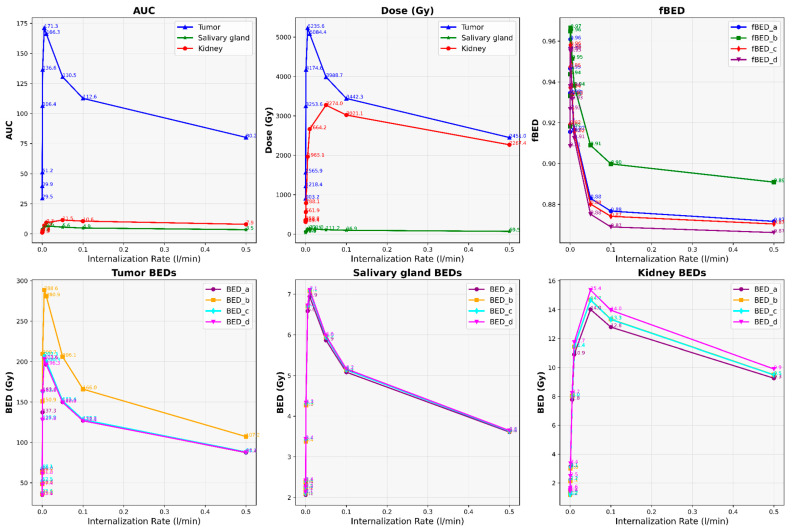
The impact of changes in internalization rate (L/min) on AUC, dose, BED, and fBED in tumors, salivary glands, and kidneys when tumor release rate is zero.

Figure 6 and Figure 7 depict the results for the association rate and internalization rate, and Supplementary Figures S6–S8 show the results for receptor density, ligand amount, and tumor volume, respectively. In the subsequent sections, we provide detailed explanations for the results of each parameter.

Figure 6 illustrates the impact of changes in the association rate (nmol/L/min) on AUC, dose, BED, and fBED in tumors, salivary glands, and kidneys when the tumor release rate is set to zero. In this section, we made the tumor release rate zero and then conducted this analysis. As the association rate increases from 0.001 to 0.1 nmol/L/min, the AUC values for the tumor show a notable rise from 3189.53 to 4190.76, indicating a higher uptake in the tumor. Similarly, the salivary gland and kidney AUC values also increase, but to a much lesser extent, suggesting a more targeted effect in the tumor. The dose to the tumor increases from 104.35 to 137.10 Gy, while doses to the salivary gland and kidney remain relatively low, showing a minimal increase from 2.79 to 4.07 Gy for the salivary gland and from 2.21 to 2.75 Gy for the kidney. BED values for the tumor also exhibited a significant rise, with BED_L increasing from 119.08 to 164.68 Gy and BED_G from 149.98 to 211.38 Gy. The corresponding BED values for the salivary glands and kidneys show a much smaller increase. The fBED values for the tumor consistently remain high, with fBED_L starting at 0.958 and showing minimal changes across the association rates, indicating a stable biological effectiveness despite the increasing association rate. In contrast, fBED values for the salivary glands and kidneys are slightly lower, reflecting the reduced impact on these organs compared to the tumor. These results suggest that optimizing the association rate can significantly enhance tumor targeting and treatment efficacy while minimizing the impact on non-target tissues, particularly when the tumor release rate is controlled.

Figure 7 illustrates the impact of changes in the internalization rate (L/min) on AUC, dose, BED, and fBED in tumors, salivary glands, and kidneys when the tumor release rate is set to zero. In this section, we made the tumor release rate zero and then conducted this analysis. As the internalization rate increased from 0.00001 to 0.5 nmol/L/min, AUC values in the tumor increased significantly from 29.55 to 171.28 before slightly declining at higher rates, reaching between 130.49 at 0.05 nmol/L/min and 80.18 at 0.5 nmol/L/min. AUC values in the salivary glands and kidneys followed a similar trend, but with smaller magnitudes, increasing from 6.57 to 11.47, respectively, at 0.01 nmol/L/min before decreasing. Dose values in the tumor rose sharply from 903.19 to 5235.57 as the internalization rate increased to 0.005 and then decreased to 2450.99 at 0.5 nmol/L/min. In the salivary glands, the dose increased from 39.51 to 131.00, while in the kidneys, it rose from 309.44 to 1965.13 at 0.005 nmol/L/min and then decreased to between 69.54 and 2267.38, respectively, at 0.5 nmol/L/min.

BED values in tumors showed a significant rise from 35.04 at the lowest internalization rate to 288.58 at 0.005 nmol/L/min, then a decline to 149.99 at 0.05 nmol/L/min. Similarly, in the salivary glands and kidneys, BED values peaked between 6.58 and 8.03, respectively, before declining at higher internalization rates. fBED values for the tumor remained relatively high across the range, starting from 0.915 at 0.00001 nmol/L/min, peaking at 0.961 at 0.0005 nmol/L/min, and decreasing to 0.871 at 0.5 nmol/L/min. Similar trends were observed in the salivary glands and kidneys, with fBED values peaking between 0.952 and 0.948 before tapering off. In summary, an intermediate internalization rate around 0.005 nmol/L/min optimizes therapeutic outcomes by maximizing AUC, dose, and BED values in tumors while maintaining lower off-target effects in the salivary glands and kidneys. This suggests that a careful balance is necessary for effective radiopharmaceutical therapy.

As observed in Supplementary Figure S6, when the release rate of the tumor is set to zero, varying the ligand amount significantly impacts the pharmacokinetic parameters across tumors, salivary glands, and kidneys. As the ligand amount increases, AUC_Tumor escalates from 558.01 at 1 nmol/L to 52,831.91 at 100 nmol/L, while AUC_Salivary and AUC_Kidney rise from 10.95 to 830.68 and 108.15 to 7628.41, respectively. Correspondingly, the dose to the tumor (D_Tumor) shows a substantial increase from 18.26 Gy at 1 nmol/L to 1728.40 Gy at 100 nmol/L, with D_Salivary and D_Kidney also escalating from 0.55 to 41.67 Gy and from 0.38 to 26.73 Gy, respectively. Similarly, BED values for the tumor also increase significantly: BED_L_Tumor rises from 18.74 at 1 nmol/L to 6284.72 at 100 nmol/L, and BED_G_Tumor increases from 19.60 to 13,281.96. BED values in the salivary glands and kidneys follow a similar trend. Notably, the fractional BED (fBED) values exhibit a consistent rise with ligand amount, with fBED_L increasing from 0.952 to 0.981 for the tumor, indicating a proportionate increase in effective dose with higher ligand amounts. These results underscore the strong correlation between ligand concentration and dose metrics, reinforcing the need for careful calibration of ligand amounts to optimize therapeutic outcomes while minimizing toxicity in non-target tissues.

### 3.3. Impact of PBPK Modeling Parameters on Time–Activity Curve Features

**Figure 8:**
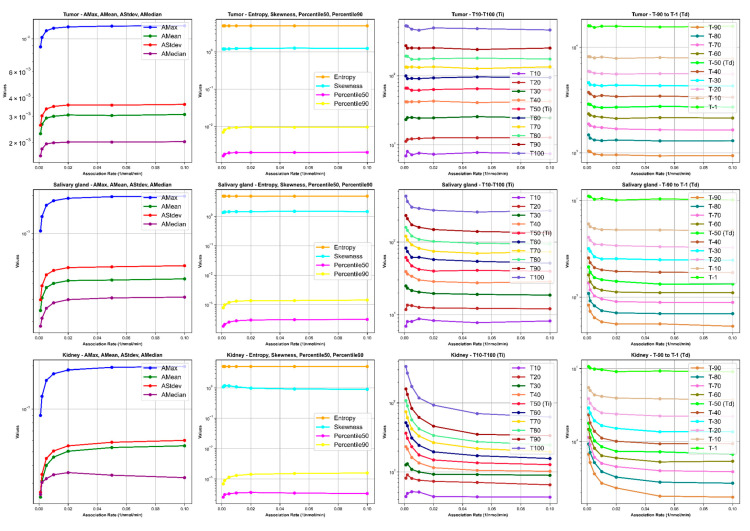
The impact of changes in association rate (L/nmol/min) on time–activity curve (TAC) features. *Ti* and *Td* are increase and decrease half-time, respectively.

**Figure 9:**
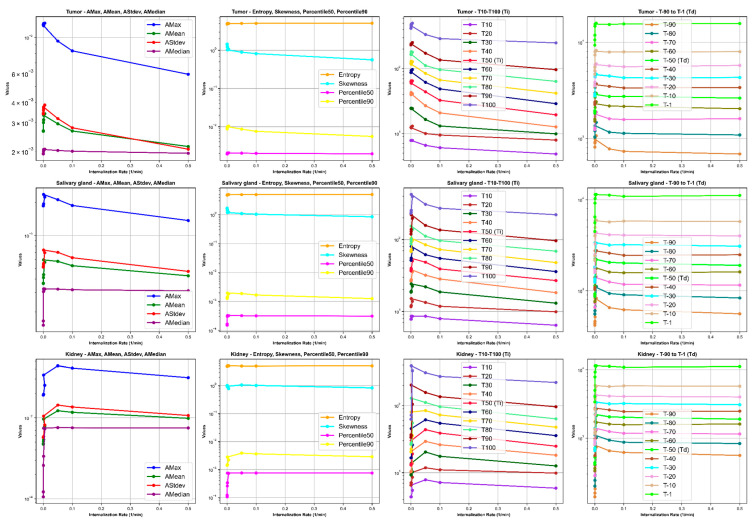
The impact of changes in internalization rate (L/min) on time–activity curve (TAC) features. *Ti* and *Td* are increase and decrease half-time, respectively.

**Figure 10:**
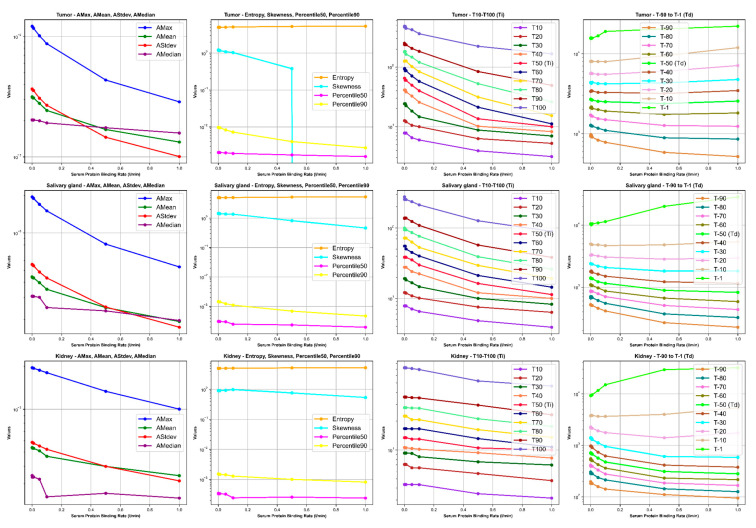
The impact of changes in serum protein-binding rate (L/min) on time–activity curve (TAC) features. *Ti* and *Td* are increase and decrease half-time, respectively.

**Figure 11:**
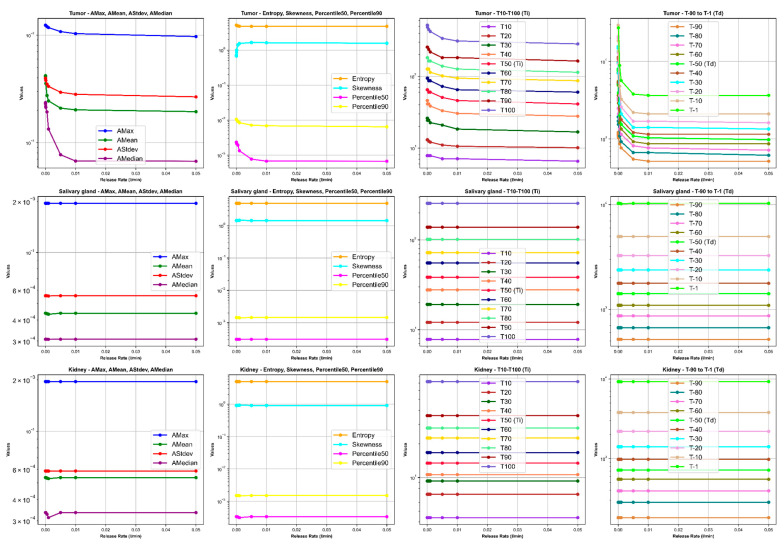
The impact of changes in tumor release rate (L/min) on time–activity curve (TAC) features. *Ti* and *Td* are increase and decrease half-time, respectively.

Figure 8, Figure 9, Figure 10 and Figure 11 illustrate the impact of changes in various parameters (association rate, internalization rate, serum protein-binding rate, and tumor release rate) on the logarithm of TAC feature values for tumors, salivary glands, and kidneys. Across all tissues, increasing these rates generally results in significant changes in TAC features. For tumors, higher rates lead to increased AMax, AMean, AStdev, and AMedian initially, followed by plateauing or decreasing trends, indicating faster uptake and clearance of the radiopharmaceutical. Entropy and skewness remain relatively stable, while Percentile50 and Percentile90 show minor variations. The times to reach the activity peaks (T10 to T100) generally decrease with increasing rates, while the times to decay (T90 to T1) significantly decline, suggesting quicker radiopharmaceutical processing. Similar but less pronounced trends are observed for salivary glands and kidneys, with slight increases or decreases in AMax, AMean, AStdev, and AMedian, stable entropy, and skewness, and moderate reductions in increasing and decreasing times. The logarithmic *y*-axis in these figures enhances the visualization of exponential changes in feature values, emphasizing the importance of optimizing these rates for effective treatment across different tissues.

Supplementary Figures S9–S11 illustrate the impact of changes in ligand amount (nmol), tumor receptor density (nmol/L), and tumor volume (L) on the logarithm of TAC feature values for tumors, salivary glands, and kidneys. Across all tissues, increasing ligand amounts result in exponential increases in features such as AMax, AMean, AStdev, and AMedian, reflecting enhanced radiopharmaceutical uptake. Entropy and skewness remain relatively stable, while Percentile50 and Percentile90 increase, indicating changes in the distribution spread. With higher receptor densities, these features initially rise rapidly and then plateau, indicating a saturation point in radiopharmaceutical uptake. Tumor volume changes lead to significant variations in TAC features, with larger volumes being generally associated with longer times to reach activity peaks (T10–T100) and shorter times to decay (T90–T1). These results highlight the importance of optimizing ligand amount, receptor density, and tumor volume to achieve effective radiopharmaceutical uptake and processing in different tissues. The logarithmic *y*-axis in these figures helps to clearly visualize the exponential changes in feature values.

### 3.4. Impact of PBPK Modeling Parameters on RBE

**Figure 12:**
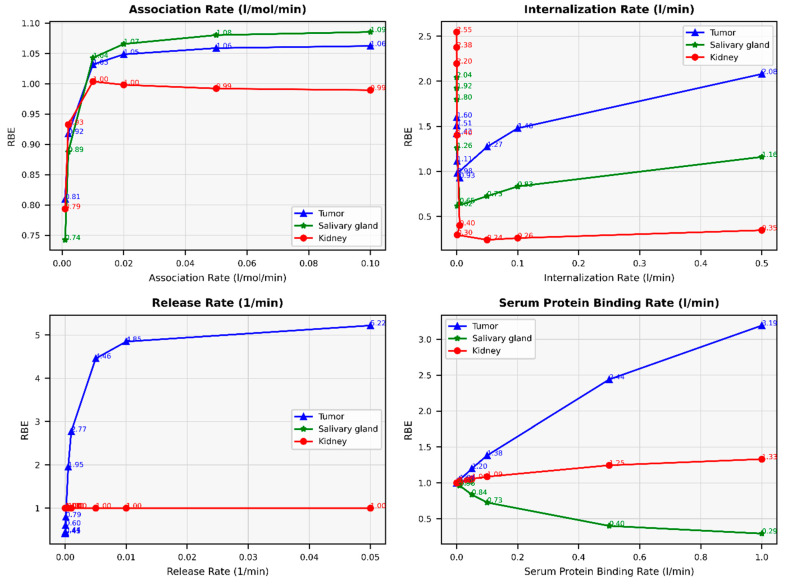
The impact of changes in association rate (L/nmol/min), internalization rate (L/min), release rate (L/min), and serum protein-binding rate (L/min) on relative biological effectiveness (RBE).

Figure 12 illustrates the impact of changes in various parameters (association rate, internalization rate, serum protein-binding rate, and tumor release rate) on the RBE. The results indicate that varying key parameters significantly impacts the relative biological effectiveness (RBE) for tumors, salivary glands, and kidneys. For the association rate, increasing from 0.001 to 0.1 L/nmol/min generally enhances the RBE for tumors (from 0.8096 to 1.0622) and salivary glands (from 0.7424 to 1.0853) while slightly decreasing it for kidneys (from 0.7937 to 0.9894). For the internalization rate, decreasing from 0.001 to 0.00001 L/min increases the RBE for all tissues, with tumors rising from 1 to 1.5980, salivary glands from 1 to 2.0436, and kidneys from 1 to 2.5467. Higher internalization rates reduce RBE, particularly for kidneys, dropping to 0.3476 at 0.5 l/min. For the tumor release rate, increasing from 0.00024 to 0.05 L/min significantly boosts the RBE for tumors from 1 to 5.2233, with minimal impact on salivary glands and kidneys, which remain around 1. Lastly, for the serum protein-binding rate, increasing from 0.00047 to 1 L/min raises the RBE for tumors from 1 to 3.1927 and kidneys from 1.0005 to 1.3313, but decreases it for salivary glands from 1 to 0.2930.

## 4. Discussion

Cancer treatment employing novel radiopharmaceuticals has witnessed significant strides in recent years. Among these, ^177^Lu-PSMA RPTs have undergone extensive evaluation in numerous clinical trials for managing prostate cancers, including metastatic castration-resistant cases [31]. The results from these trials have demonstrated highly favorable clinical outcomes, notably contributing to increased overall survival [32]. However, the efficacy of these therapeutic modalities, particularly in the context of managing challenging scenarios such as metastatic castration-resistant cases, transcends singular determinants. Instead, it intricately depends on a multifaceted interplay of physical, biological, physiological, and pharmacological factors [33]. The optimization of these factors is paramount to achieving optimal outcomes, characterized by heightened tumor control and mitigated complications in normal tissues.

As elucidated by our research findings, the utilization of model-informed RPTs, notably incorporating PBPK modeling, is a promising avenue for refining treatment strategies through optimization and personalization. Our investigative analyses pave the way for new possibilities, specifically in the realm of predictive modeling, to tailor treatments based on individual characteristics. Within the scope of this study, we delved into the examination of how PBPK model parameters influence measured outcomes, including both physical and biological doses. Our analysis revealed that the estimation and optimization of these parameters can exert profound impacts on such measures. These insights can help advance research and refine clinical workflows, underlining the significance of understanding and manipulating PBPK model parameters in the pursuit of enhanced precision in therapeutic approaches. As an example, one can consider the feasibility of analyses for dynamic Positron Emission Tomography (PET) images obtained pre-therapy for different radiopharmaceuticals. One would be able to discern the parameters influencing PET measures, as alterations induced by specific PBPK parameters on PET scans imply the sensitivity of the imaging modality to those factors. Subsequently, if a parameter significantly influences both PET measures and subsequent therapeutic doses, it becomes a valuable candidate for enhanced dose estimation through predictive modeling. This iterative process, which we are actively investigating, harnesses the interplay between parameter sensitivity in PET scans and their impact on therapeutic dosage, providing a pathway for refining dose estimations based on pre-therapy dynamic PET scans.

In this study, we identified various physical, physiological, and pharmacokinetic parameters integrated into our PBPK model that significantly influence the therapeutic dose in both tumorsnd normal organs. Specifically, we explored the impact of changes in pharmacokinetic parameters such as the of the ^177^Lu-PSMA-binding association rate, internalization rates into normal and tumor cells, binding rates to serum proteins, and release rates from cells. Additionally, we investigated physiological factors like tumor receptor density and tissue volume, along with physical parameters such as ligand amount. Our investigation uncovered important findings. To comprehensively interpret these outcomes, it is imperative to emphasize the substantial impact of influential factors like S-values and the repair constant (µ). These factors play a critical role in shaping the overall outcomes, especially as we calculate important measures like dose and BED. It is essential to precisely define everything related to these measures. In particular, the specific values of S-value, µ, *Ti*, *Td*, and decay constants are crucial. Notably, ongoing debates surround S and µ values, necessitating further exploration, especially in their application within clinical settings. The dynamic nature of these considerations emphasizes the ongoing need for research to uncover their full implications and enhance their practical integration into medical applications. In order to enhance the robustness and applicability of our findings, a more in-depth investigation into the nuances and potential variations in these factors in clinical contexts is warranted.

In this study, we selected the key parameters identified in previous research because they have significant roles in patient response, influencing both the physical and biological dose delivered to the tumor and normal tissues, and ultimately the clinical outcome [21,34]. We focused on the association rate, internalization rate, serum protein-binding rate, release rate, receptor density, ligand amount, and tumor volume because they directly impact the effectiveness of radiopharmaceutical therapy. The association and internalization rates affect the initial uptake and retention of the radiopharmaceutical in the tumor. The serum protein-binding rate impacts drug availability and distribution, while the release rate affects the duration of the therapeutic effect. Receptor density influences binding efficiency, and ligand amount directly impacts the dose delivered. Tumor volume affects the distribution and penetration of radiopharmaceuticals. Further studies are needed to explore additional parameters such as the dissociation constant, serum flow rate, permeability surface area, kidney glomerular filtration rate, and the inclusion of other normal organs and their parameters, as well as other patient-specific factors to optimize dose distribution and clinical outcomes. In addition, further studies are needed to investigate how drug metabolism varies with different parameters. Understanding these variations will provide deeper insights into optimizing radiopharmaceutical therapy and improving clinical outcomes. Incorporating detailed analyses of metabolic pathways and their interactions with key parameters will be crucial for refining treatment strategies.

Our research results bring to light the significant influence of crucial parameters, namely tumor volume, release rate, receptor density, and the amount of administrated radioligands on measured outcomes such as the area under the time–activity curve, dose, and BED. This observation aligns well with supporting evidence from previous clinical and research studies [21]. A key revelation in our findings is the distinct manifestation of the tumor sink effect. Specifically, as tumor volume or receptor density increases, there is a notable intensification in the distribution of the radiopharmaceutical by the tumor. This phenomenon aligns with clinical manifestations observed in RPTs [35,36]. In the practical realm of RPTs, this heightened absorption by the tumor is pivotal, as it effectively reduces the dose delivered to normal organs. These insights help us better understand how these factors influence the effectiveness and safety of RPTs, highlighting the crucial role of the tumor sink effect in improving treatment outcomes.

Even though tumor physiological factors such as receptor density and volume play a crucial role in altering the physical/biological dose, other pharmacokinetic parameters for both tumor and normal tissues have a substantial impact, as evidenced by our observations in this study. Since our PBPK model is based on the physio(bio)logical process of PSMA binding/internalization/degradation [37], each related kinetic parameter for PSMA-positive organs (here: tumor, kidneys, and salivary gland) impacts the dose (D and BED). For instance, we noted variations in the uptake (AUC) of the kidney compared to that of the tumor and salivary glands under different parameter settings, such as association, internalization, and release rates. This discrepancy can be attributed to the kidney’s pivotal role in clearing PSMA from the body. Notably, the kidney boasts a significantly larger volume (0.311 L) compared to the default volume of the tumor (0.01 L). Nevertheless, the outcomes diverge when considering metrics like dose, BED, and fBED.

In our research, we employed four distinct BED models—Lea–Catcheside, a simplified Lea–Catcheside, Kalogianni et al., and Howel et al. to analyze radiobiological outcomes using data from the time–activity curve (TAC). These models incorporate crucial parameters such as initial dose rate (r0), increasing and decreasing half-lives (*Ti* and *Td*), effective decay (λ), repair constant (µ), and effective clearance and uptake constants (λe and λeu). Our findings demonstrate that variations in these parameters significantly influence clinical outcomes, highlighting the necessity of advanced modeling to accurately represent dose–response relationships in radiopharmaceutical therapy (RPT). While simpler BED models have been used for RPTs, our advanced models capture the complex exponentially changing dose patterns essential for understanding radiation-induced resistance or sensitivity. This underscores the critical role of detailed pharmacokinetic parameters in enhancing the precision and effectiveness of personalized RPT, an area still evolving compared to external beam radiotherapy. It is crucial to emphasize that dose–response modeling is integral to radiation oncology. While numerous models have been proposed in external beam radiotherapy [38], they are relatively new and actively researched in the realm of personalized RPTs. Our study highlights the impact of various pharmacokinetic parameters on TAC behavior, underscoring the necessity of their direct inclusion in modeling efforts.

As a part of our work, we discovered the profound significance of the tumor release rate in the modeling of ^177^Lu-PSMA. Our findings indicate that a deliberate reduction in the release rate of the radiopharmaceutical leads to an augmented accumulation of physical and biological doses within the tumor compared to the standard scenario. While this investigation is theoretical in nature, its implications can be experimentally tested through preclinical studies. Potential avenues include modifying radiopharmaceuticals, enhancing their binding to specific materials to prolong cellular retention, and exploring strategies to effectively curtail the release rate. These practical tests could offer valuable insights into optimizing the therapeutic potential of ^177^Lu-PSMA.

In this study, we introduced the concept of fBED, for the first time to our knowledge, as a unique metric to assess RPT plans. Building on previous research that highlighted the potential criticality of doses received by PSMA-positive normal organs, such as the kidneys and salivary glands, predicting the dose for these organs emerges as a crucial step in personalizing the therapy [21,39]. Our fBED incorporates Biologically Effective Dose considerations for both tumors and normal organs, providing a radiobiological-guided measure for RPT. While the predictive efficacy of such measures should be rigorously assessed in clinical or preclinical studies, within the simulation realm, its application proves to be intriguing. Moreover, this metric allows for refinement by incorporating weighting factors for both target and non-target organs, enabling the evaluation of different organs. This approach holds promise for future optimizations in RPTs. In prior analogous studies, the term “overall biologically effective dose (oBED)” was introduced, focusing on multiple tumors [40]. In a unique part of our study, we focused on extraction of novel features from TACs, marking the first instance of exploring how these features undergo alterations in response to changes in various pharmacokinetic, physical, and physiological parameters. Although the prediction power of these features has not been previously investigated, they can open a new area of research in the realm of RPTs. The radiobiological features, specifically *Ti* and *Td*, take center stage in our investigation due to their pronounced significance. The dynamic alterations in *Ti* and *Td*, influenced by modeling parameters, hold particular interest, as they potentially unveil crucial biological implications, as highlighted in the work of Solanki et al. This emphasis on radiobiological features underscores their potential as key indicators, shedding light on the nuanced intricacies of the biological responses under diverse modeling conditions [18].

As part of this study, we calculated the relative biological effectiveness (RBE) and examined how varying key parameters influence treatment outcomes in radiopharmaceutical therapy. The RBE values are related to changes in these parameters compared to the default values obtained from the literature, and our model has been validated based on these default values. Our findings demonstrate that optimizing the association rate, internalization rate, tumor release rate, and serum protein-binding rate can significantly impact the RBE for tumors, salivary glands, and kidneys. Increasing the association and internalization rates generally improves the RBE for tumors, suggesting better uptake and retention of the radiopharmaceutical. However, higher internalization rates may reduce effectiveness in kidneys, indicating a need for balanced optimization. The tumor release rate shows a strong positive correlation with tumor RBE, emphasizing its critical role in therapeutic effectiveness. Conversely, higher serum-protein binding rates enhance tumor and kidney RBE but reduce it for salivary glands, suggesting differential impacts on various tissues. Further studies should explore additional parameters and their interactions to refine dose distribution and clinical outcomes.

While our model has undergone validation with clinical data, the translation and integration of the model into routine clinical practice demands thoughtful consideration. Despite extensive exploration of PBPK modeling by various researchers with numerous suggested parameters, our results underscore the critical importance of accurate parameter estimation for individual patients to personalize therapy effectively. Given the cyclical nature of RPTs, there is a pressing need for dynamic PBPK modeling and parameter estimation. This is crucial for capturing therapy-induced kinetic changes and incorporating them into the planning of subsequent therapy cycles. In the contemporary landscape, integration of dynamic imaging techniques and innovative approaches, such as total body PET scanners and artificial intelligence-based methods, holds significant promise in advancing the precision and efficacy of personalized therapy in the clinical setting. Furthermore, in the application of PBPK modeling in the field of RPT, it is essential to highlight that many existing models employ ODEs to represent radiopharmaceutical distribution within organs. A notable research venue includes the development of models utilizing partial differential equations (PDEs), offering a more realistic representation of radiopharmaceutical distribution by accounting for spatiotemporal heterogeneities of radiopharmaceutical distributions [23]. These models remain to be fully evaluated and compared and contrasted against PBPK/ODE approaches to assess their suitability for routine deployment.

## 5. Conclusions

Our study systematically evaluates the influence of various PBPK parameters on AUC, dose, BED, and fBED outcomes, focusing on association rate, internalization rate, serum release rate, serum protein-binding rate, and tumor volume. The results show that higher association rates significantly enhance radiation delivery and effectiveness in tumors, with minimal impact on non-target organs. Increasing internalization rates reduce radiopharmaceutical uptake in tumors and kidneys, while the salivary gland remains largely unaffected. Elevating release rates decreases AUC and dose across all organs, emphasizing the importance of tumor release rate in dose optimization. Higher serum protein-binding rates decrease AUC and dose for tumors, kidneys, and salivary glands. Increasing tumor receptor density significantly improves uptake and dose delivery to tumors while minimally affecting non-target organs. Additionally, larger ligand amounts and tumor volumes enhance uptake and dose delivery to tumors. Our analysis using four different BED models (simplified Lea–Catcheside, Kalogianni et al., original Lea–Catcheside, and Howell et al.) revealed similar trends across different parameters. Furthermore, our findings highlight the importance of time–activity curve features such as AMax, AMean, AStdev, AMedian, *Ti*, and *Td* in predictive modeling and treatment efficacy. This approach provides a pathway to systematically test diverse RPT scenarios and assess the impact of various radiopharmaceuticals. By incorporating patient-specific parameters and responses, a more accurate representation of the complexities involved in RPT can be captured, paving the way for the exploration of optimal therapeutic strategies and enhancing overall treatment efficacy. This comprehensive analysis underscores the potential of model-informed personalization in optimizing RPTs, reducing toxicities, and enhancing therapeutic efficacy.

## Figures and Tables

**Table 1:** The range of parameters studied in this work. Definition, values, and units are shown.

Parameter	Definition	Assigned Values	Unit
Association rate	Rate at which radioligand binds to target receptors.	0.001, 0.002, 0.005, 0.01, 0.02, 0.05, 0.1	L/nmol/min
Internalization rate	Rate at which radioligand is taken into cells.	0.00001, 0.00005, 0.0001, 0.0005, 0.001, 0.005, 0.01, 0.05, 0.1, 0.5	L/min
Serum protein-binding rate	Rate at which radioligand binds to serum proteins.	0.0001, 0.0005, 0.001, 0.005, 0.01, 0.05, 0.1, 0.5, 1	L/min
Release rate	Rate at which radioligand is released from the cells.	0, 0.00001, 0.00002, 0.00005, 0.0001, 0.0002, 0.00025, 0.0003, 0.0005, 0.001	L/min
Receptor density	Concentration of target receptors on tumor cells.	1, 5, 50, 100, 500, 1000, 2000, 5000	nmol/L
Ligand amount	Quantity of injected radioligand ligands.	1, 5, 10, 25, 50, 100,	nmol
Tumor volume	Size or volume of the tumor being treated.	0.001, 0.005, 0.01, 0.05, 0.1, 0.5, 1	L

**Table 2:** Features extracted from the time–activity curves (TACs).

Feature	Definition
A_max_	The maximum concentration of the radiopharmaceutical in the tissue.
A_mean_	The mean concentration of the radiopharmaceutical in the tissue.
A_Stdev_	The standard deviation of the concentration of the radiopharmaceutical in the tissue.
A_median_	The media concentration of the radiopharmaceutical in the tissue.
Skewness	A measure of the asymmetry in the distribution of the concentration values within the time–concentration profile of the radiopharmaceutical, indicating the dominance of higher or lower concentrations.
Entropy	A measure of the unpredictability or randomness in the distribution of the concentration values over time in the radiopharmaceutical profile, reflecting the complexity and diversity of the pattern.
Percentile_50_	The value at which 50% of the activity measurements lie below it and 50% lie above it.
Percentile_90_	The value below which 90% of the activity measurements fall.
T_i_	The approximate time required for the radiopharmaceutical dose rate to increase to one-half of its maximum value.
T_d_	The approximate time required for the radiopharmaceutical dose rate to decrease to one-half of its maximum value.
Increasing times (T_10_–T_90_)	The time at which the activity first reaches 10–90% of its maximum value during the initial increase.
Decreasing times (T_−10_–T_−90_)	The time at which the activity decreases to 10–90% of its maximum value during the decay phase.

## Data Availability

Data are available upon reasonable request.

## Supplementary Materials

The following supporting information can be downloaded at: https://www.mdpi.com/article/10.3390/cancers16183120/s1, Figure S1: Our PBPK model structure, compartments, and sub-compartments; Figure S2. Dose-rate pattern characterized by an initial exponential increase in dose rate, followed by an exponential decrease in dose rate; Figure S3. Dose rate and r0 values; Figure S4. The impact of changes in Ligand Amount (nmol) on AUC, Dose, BED and fBED in tumor, salivary gland, and kidney; Figure S5. The impact of changes in Tumor Volume (l) on AUC, Dose, BED and fBED in tumor, salivary gland, and kidney; Figure S6. The impact of changes in Ligand Amount (nmol) on AUC, Dose, BED and fBED in tumor, salivary gland, and kidney, when tumor release rate is zero; Figure S7. The impact of changes in Tumor Receptor Density (nmol/l) on AUC, Dose, BED and fBED in tumor, salivary gland, and kidney, when tumor release rate is zero; Figure S8. The impact of changes in Tumor Volume (l) on AUC, Dose, BED and fBED in tumor, salivary gland, and kidney, when tumor release rate is zero; Figure S9. The impact of changes in Ligand Amount (nmol) on time activity curve (TAC) features. Ti and Td are increase and decrease half-time respectively; Figure S10. The impact of changes in Tumor Receptor Density (nmol/l) on time activity curve (TAC) features. Ti and Td are increase and decrease half-time respectively; Figure S11. The impact of changes in Tumor Volume (l) on time activity curve (TAC) features. Ti and Td are increase and decrease half-time respectively; Table S1. Model parameters for default tumor and normal organs; Table S2. Specific Values used in this study.
