# Supplementary material for: Model-Informed Radiopharmaceutical Therapy Optimization: A Study on the Impact of PBPK Model Parameters on Physical, Biological, and Statistical Measures in 177Lu-PSMA Therapy
Source: Cancers (Basel). 2024 Sep 10;16(18):3120. doi: 10.3390/cancers16183120 (PMC11430653; doi:10.3390/cancers16183120)
Supplement: Supplementary file 1 [file cancers-16-03120-s001.zip › Supplementary Texts.pdf]

Supplementary Texts

The detailed parameters used in the work.

In the following section, we have provided detailed parameters used in this work to ensure that readers can accurately reproduce the results.

Association Rate

The association rate, often denoted as (k_{on}) represents the speed at which a radioligand binds to its target receptors on the cell surface (1). It is a critical parameter in modeling the pharmacokinetics of radioligands as it directly influences the binding affinity and distribution of the ligand in the body. Higher association rates can enhance the likelihood of the radioligand binding to the receptor, thereby improving the targeting efficiency of the radioligand. This rate can be affected by factors such as ligand structure, receptor availability, and the physical properties of the surrounding medium. The assigned values for the association rate range from 0.001 to 0.1 l/nmol/min, reflecting various binding efficiencies under different biological conditions.

Internalization Rate

The internalization rate, represented as (λ_{int}) refers to the rate at which the receptor-bound radioligand is taken up into the cells. This process is crucial in determining the intracellular accumulation of the radioligand, which can significantly impact the therapeutic or diagnostic outcomes, particularly in the context of targeted radiotherapy (2). An elevated internalization rate may lead to increased cytotoxicity or improved imaging contrast due to greater intracellular retention of the radioligand. The assigned values for the internalization rate range from 0.00001 to 0.5 l/min, providing a comprehensive range to account for varying receptor-mediated endocytosis efficiencies.

Serum Protein Binding Rate

The serum protein binding rate, often denoted as (k_{pr}) defines the extent to which a radioligand binds to serum proteins in the bloodstream (3). This binding affects both the bioavailability and clearance rate of the radioligand, influencing its overall distribution and half-life in the body. High serum protein binding can lead to reduced radioligand availability for targeting, thus diminishing its efficacy in reaching the intended site of action. The values assigned to the serum protein binding rate range from 0.0001 to 1 1/min, capturing different levels of protein interaction, which can be influenced by the radioligand's physicochemical properties and the patient's physiological state.

Release Rate

The release rate denoted as (λ_{rel}) describes the speed at which the radioligand dissociates from the cells after internalization (4). This parameter is particularly important in determining the kinetics of radioligand release from targeted cells back into the extracellular space or circulation. A low release rate suggests prolonged intracellular retention, which may be desirable for therapeutic purposes, whereas a high release rate might indicate rapid clearance, beneficial for diagnostic

imaging where rapid washout is needed. The assigned values for the release rate range from 0 to 0.001 1/min, accommodating both rapid and slow dissociation scenarios.

Receptor Density

Receptor density often denoted as (R_{Tu}) reflects the concentration of target receptors available on the surface of tumor cells (5). It is a crucial determinant of the binding potential and uptake of the radioligand. Higher receptor densities generally lead to greater radioligand accumulation within the tumor, enhancing both therapeutic efficacy and imaging contrast. The assigned values for receptor density range from 1 to 5000 nmol/l, covering a spectrum from low to high receptor-expressing tumors, and thus offering insight into different tumor biology scenarios [Reference].

Ligand Amount

The ligand amount represents the total quantity of the radioligand injected into the patient, which directly affects the radioligand's concentration in systemic circulation and its subsequent biodistribution. Adjusting the ligand amount can help optimize the balance between effective targeting of tumor tissues and minimizing off-target effects or toxicity to normal tissues. The assigned values range from 1 to 100 nmol, providing a range that accommodates varying dosage levels required for different therapeutic or diagnostic protocols.

Tumor Volume

Tumor volume is a critical factor in assessing the pharmacokinetics and therapeutic efficacy of a radioligand. Larger tumor volumes may require well enough doses or longer circulation times for adequate radioligand delivery, whereas smaller tumors might be targeted more efficiently with lower doses. The assigned values for tumor volume range from 0.001 to 1 l, reflecting the diversity of tumor sizes that can be encountered in clinical practice, from small lesions to large, bulky masses.

References

1. Kletting P, Schuchardt C, Kulkarni HR, Shahinfar M, Singh A, Glatting G, et al. Investigating the Effect of Ligand Amount and Injected Therapeutic Activity: A Simulation Study for 177Lu-Labeled PSMA-Targeting Peptides. PLoS One. 2016 Sep 9;11(9):e0162303.

2. Capasso G, Stefanucci A, Tolomeo A. A systematic review on the current status of PSMA-targeted imaging and radioligand therapy. Eur J Med Chem. 2024 Jan 5;263(115966):115966.

3. Umbricht CA, Benešová M, Schibli R, Müller C. Preclinical Development of Novel PSMA-Targeting Radioligands: Modulation of Albumin-Binding Properties To Improve Prostate Cancer Therapy. Mol Pharmaceutics. 2018 Jun 4;15(6):2297–306.

4. Piranfar A, Soltani M, Kashkooli FM, Uribe CF, Rahmim A. Spatiotemporal modeling of radiopharmaceutical transport in solid tumors: Application to 177Lu-PSMA therapy of prostate cancer. Comput Methods Programs Biomed. 2024 Mar 1;245:108004.

5. Hardiansyah D, Guo W, Kletting P, Mottaghy FM, Glatting G. Time-integrated activity coefficient estimation for radionuclide therapy using PET and a pharmacokinetic model: A simulation study on the effect of sampling schedule and noise. Med Phys. 2016 Sep;43(9):5145.
