# Supplementary material for: Model-Informed Radiopharmaceutical Therapy Optimization: A Study on the Impact of PBPK Model Parameters on Physical, Biological, and Statistical Measures in 177Lu-PSMA Therapy
Source: Cancers (Basel). 2024 Sep 10;16(18):3120. doi: 10.3390/cancers16183120 (PMC11430653; doi:10.3390/cancers16183120)
Supplement: Supplementary file 1 [file cancers-16-03120-s001.zip › Supplementary_Figures.pdf]

Supplementary Figures

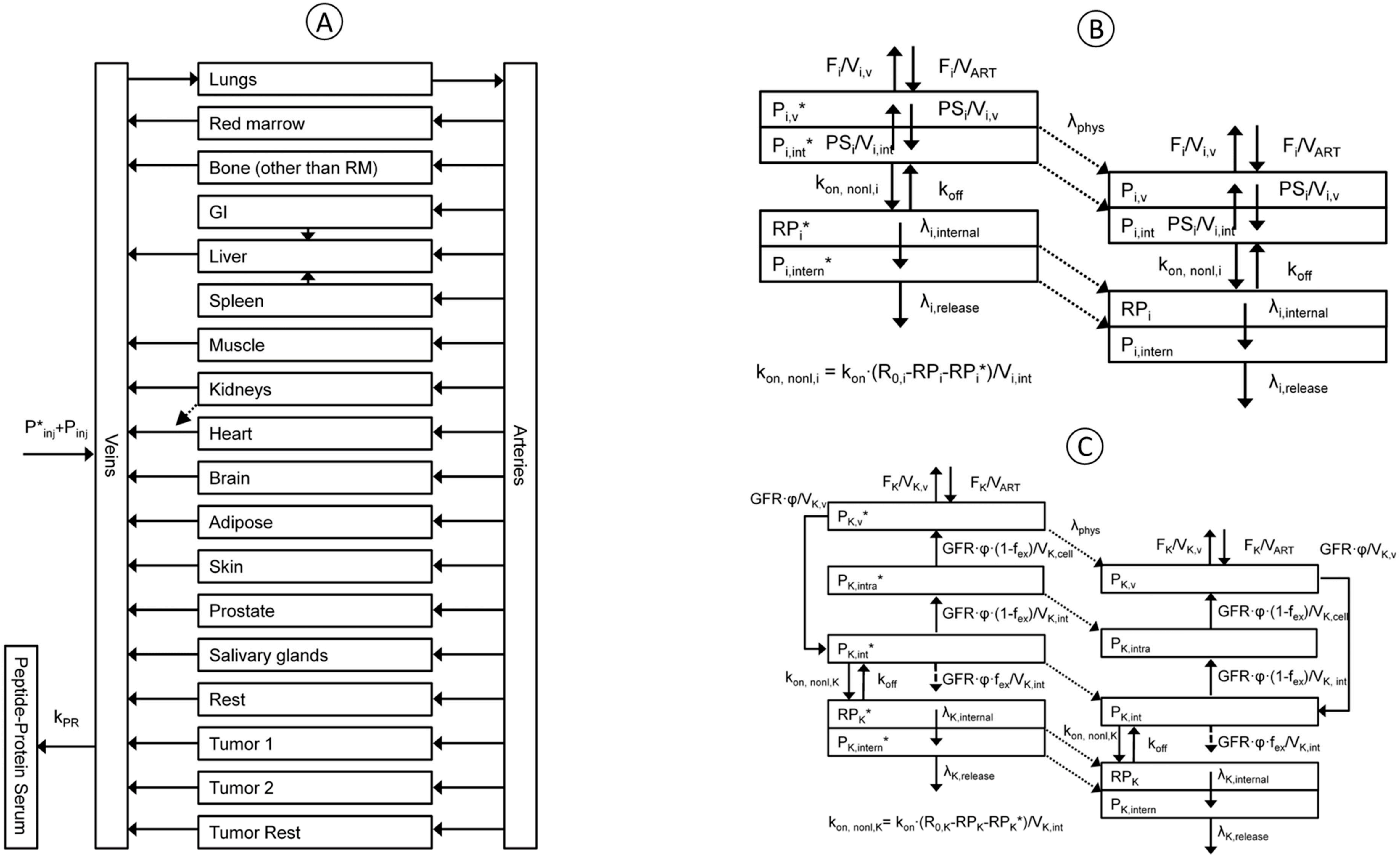

D

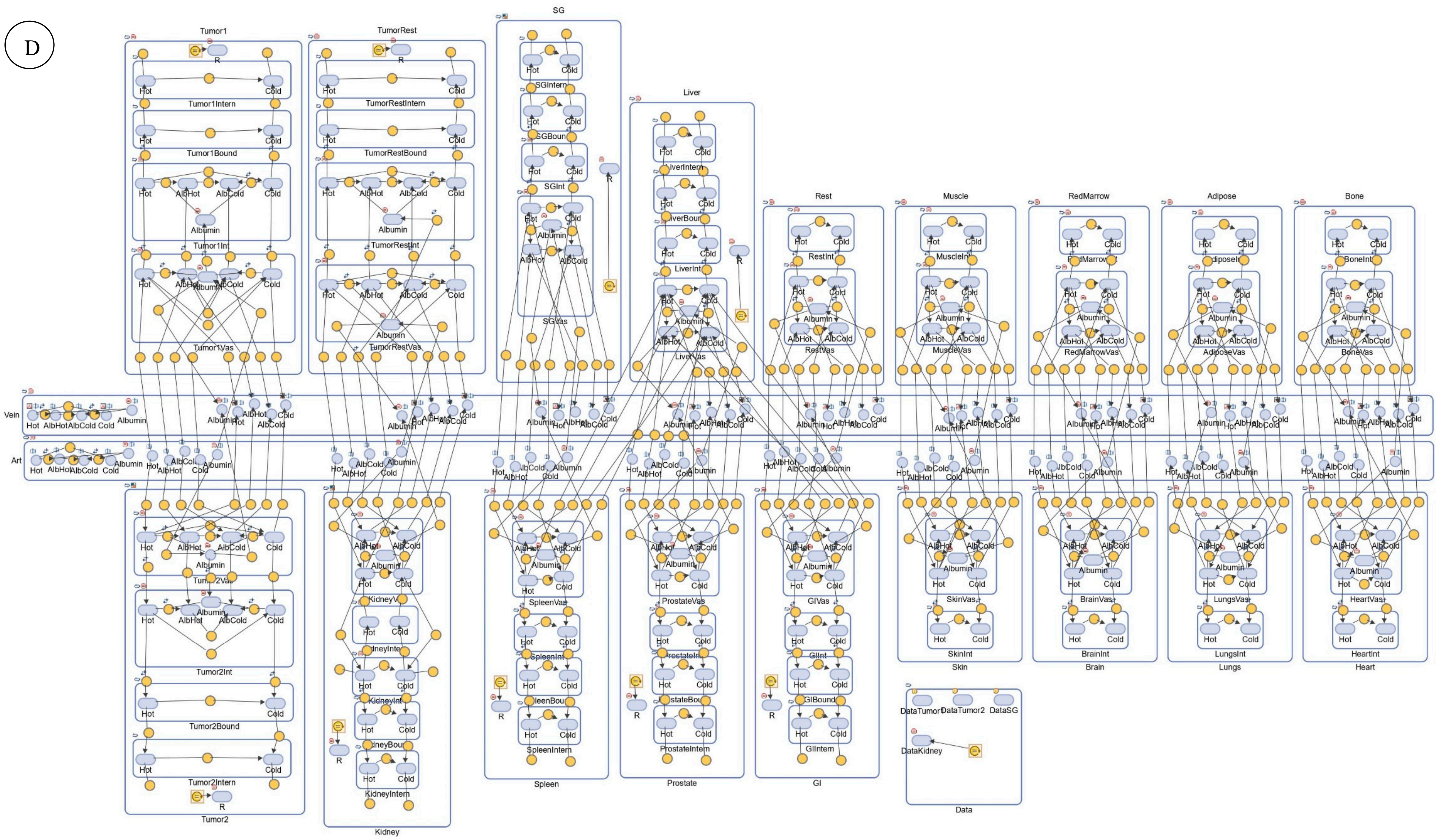

Figure S1. Our PBPK model structure, compartments, and sub-compartments. A: The model features interconnected rectangular compartments representing organs, with a "Peptide-Protein serum" compartment for bound peptides. Only veins are linked to this compartment to simplify the model, and the fitting process considers organ-specific fractions. B: Schematic representation of the integrated model for peptide dynamics in B1 tissues, encompassing GI, spleen, prostate, salivary glands, and tumor. The system involves labeled (*) and unlabeled peptide compartments, interconnected through competition for free binding sites (kon,nonl,i) and physical decay (λphys). Uniform physiological parameters are assumed for labeled and unlabeled substances. Key factors include dissociation rate (koff), peptide transport via serum flow to and from organs, PSMA-specific bound peptide (RPi), free peptide in vascular and interstitial spaces (Pi,v and Pi,int), permeability surface area product (PSi), and internalization/release rates (λi,int and λi,release). C) Kidney modelling in our PBPK model: Peptide transport involves serum flow to the vascular compartment followed by filtration into the interstitial region. Unspecific uptake mechanisms are represented by flow GFR·φ·(1-fex) in and out of kidney cells, with measured Glomerular Filtration Rate (GFR). D. The structure of PBPK model as implemented in SimBiology MATLAB.

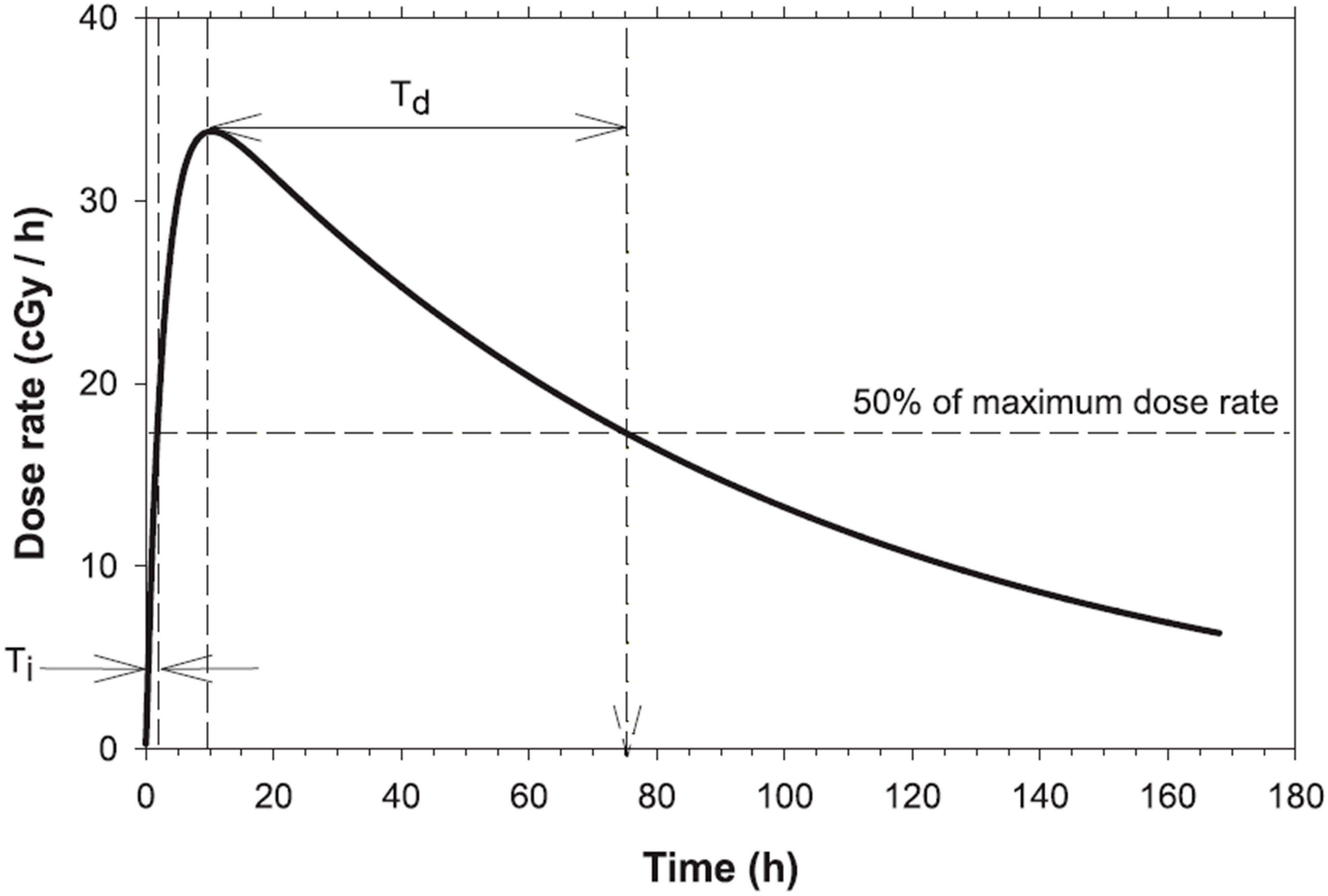

Figure S2. Dose-rate pattern characterized by an initial exponential increase in dose rate, followed by an exponential decrease in dose rate. The dose rate increase half-time Ti is the time required for dose rate to increase to about one-half of its maximum value. The dose rate decrease halftime Td is the time required for dose rate to decrease to about one-half of its maximum value. Adapted form Solanki et al. 2017 (1).

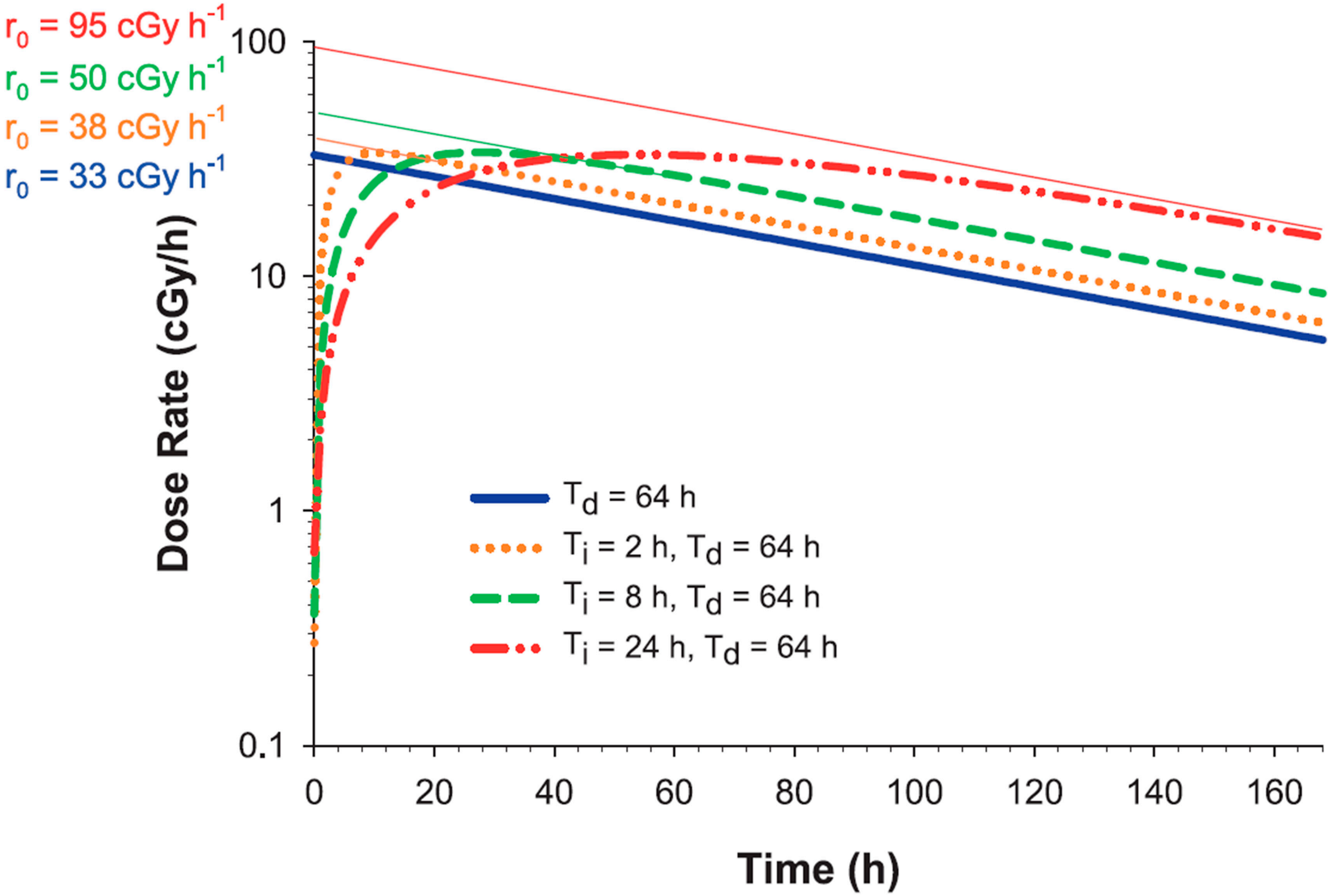

Figure S3. Dose rate and r0 values. Adapted form Solanki et al. 2017 (1).

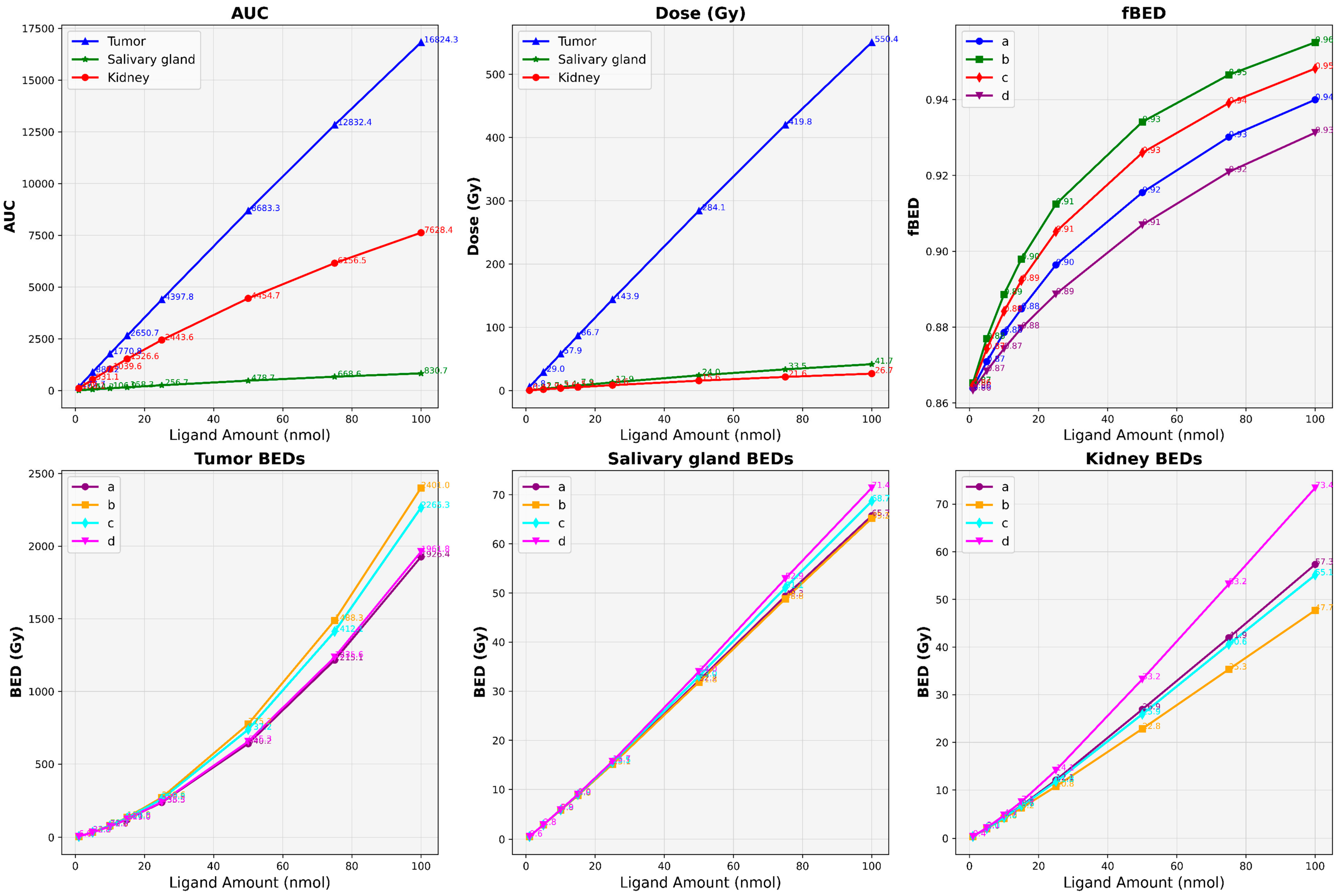

Figure S4. The impact of changes in *Ligand Amount (nmol)* on AUC, Dose, BED and fBED in tumor, salivary gland, and kidney

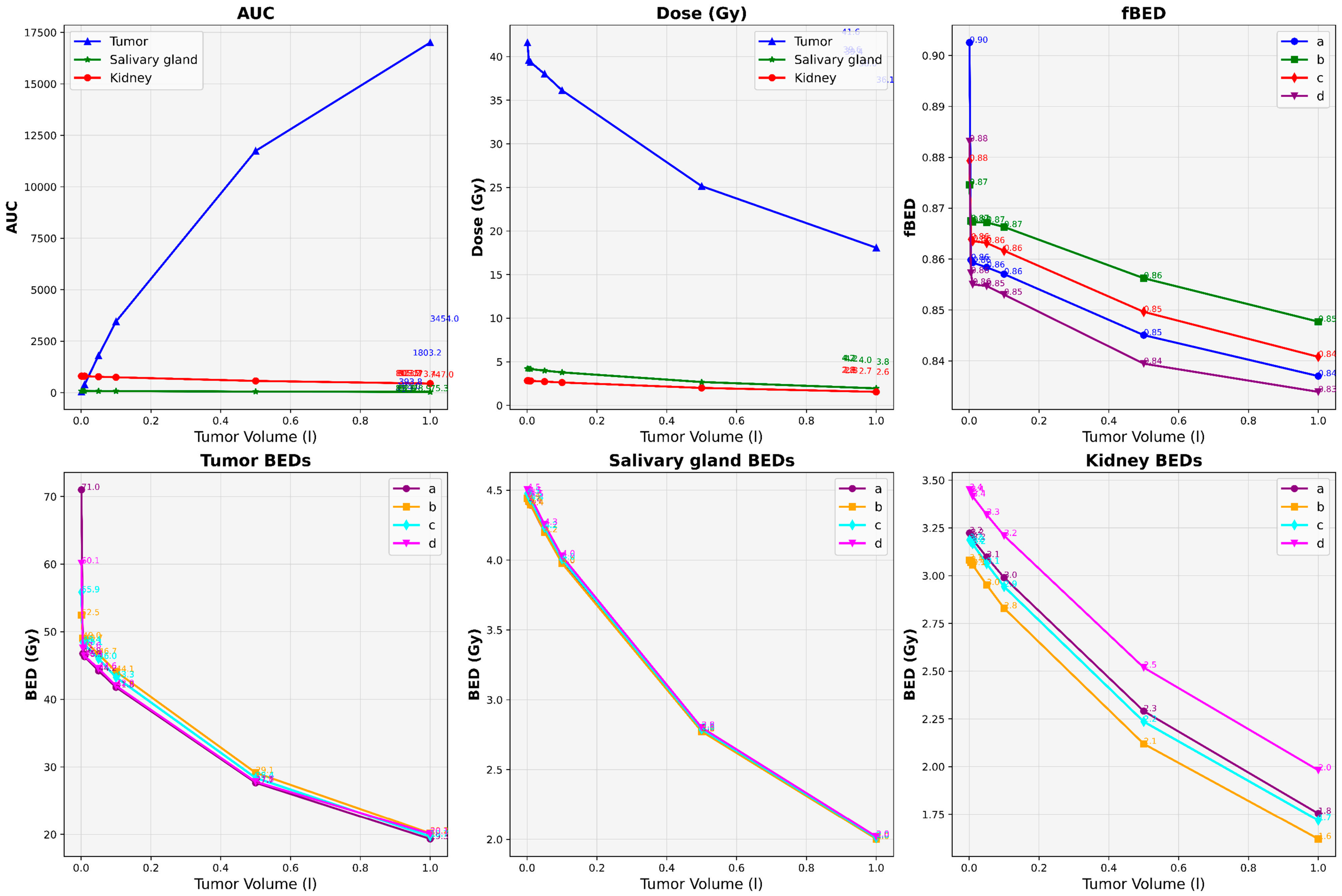

Figure S5. The impact of changes in *Tumor Volume* (l) on AUC, Dose, BED and fBED in tumor, salivary gland, and kidney

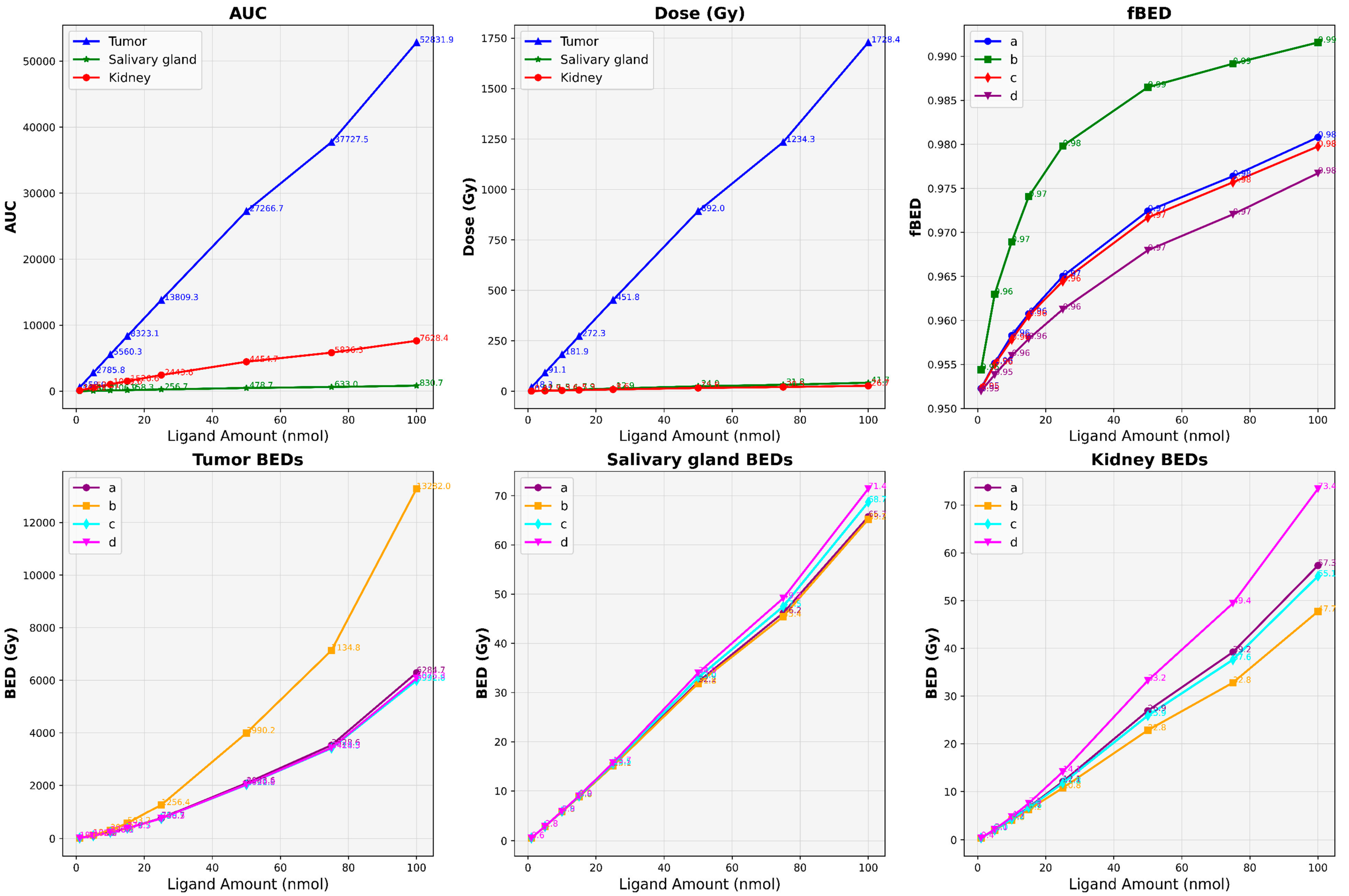

Figure S6. The impact of changes in *Ligand Amount* (nmol) on AUC, Dose, BED and fBED in tumor, salivary gland, and kidney, when tumor release rate is zero.

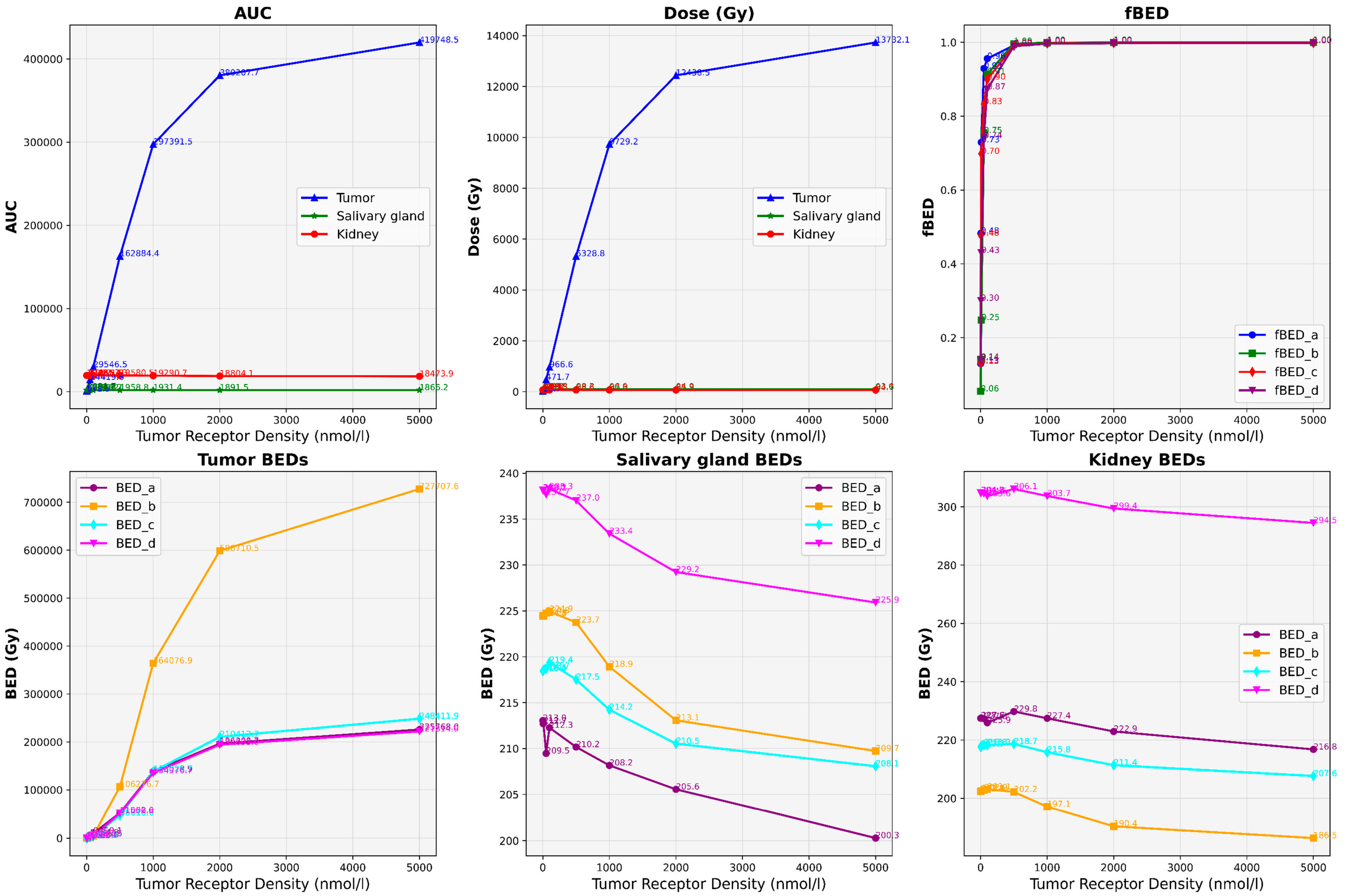

Figure S7. The impact of changes in *Tumor Receptor Density (nmol/l)* on AUC, Dose, BED and fBED in tumor, salivary gland, and kidney, when tumor release rate is zero.

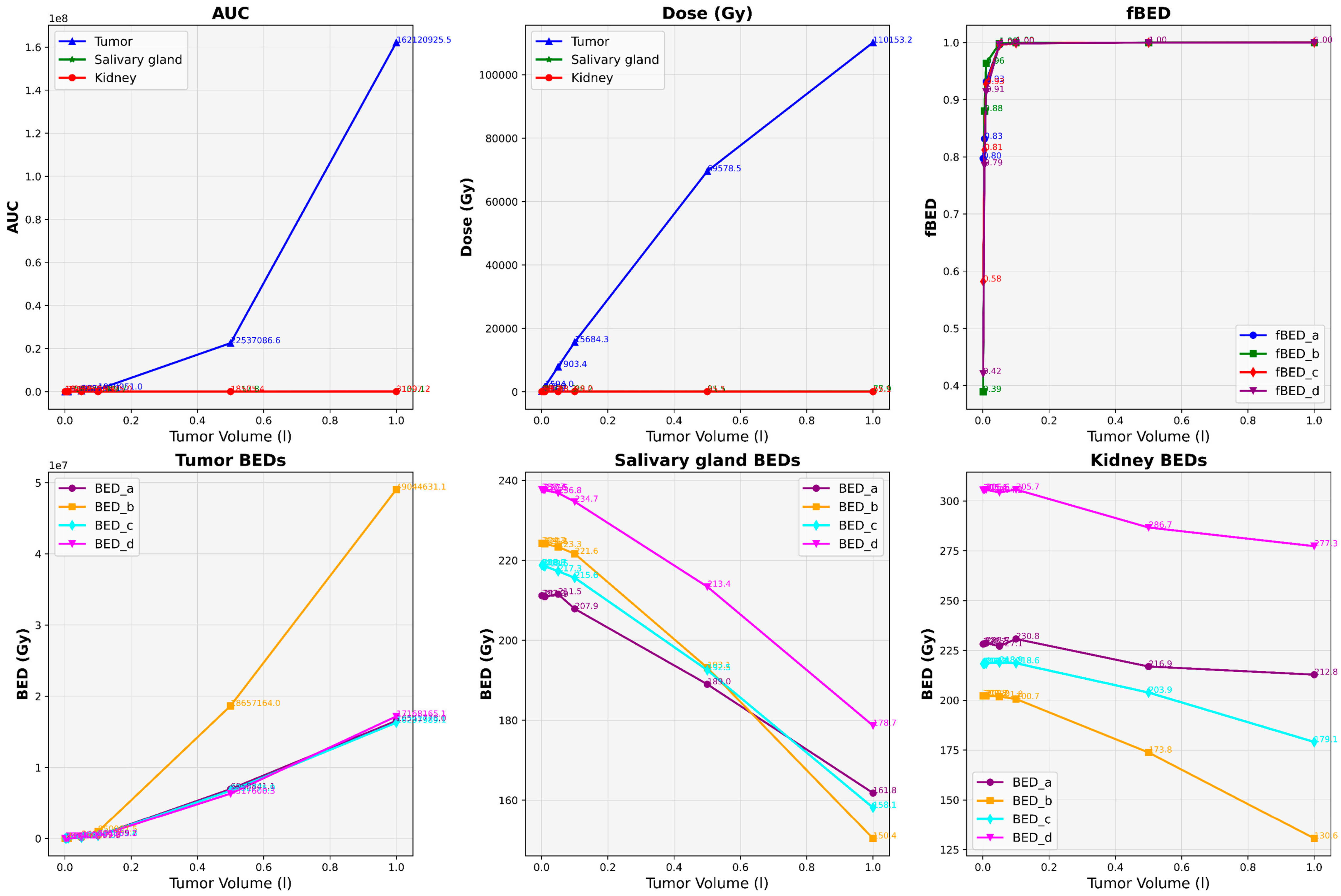

Figure S8. The impact of changes in *Tumor Volume (l)* on AUC, Dose, BED and fBED in tumor, salivary gland, and kidney, when tumor release rate is zero.

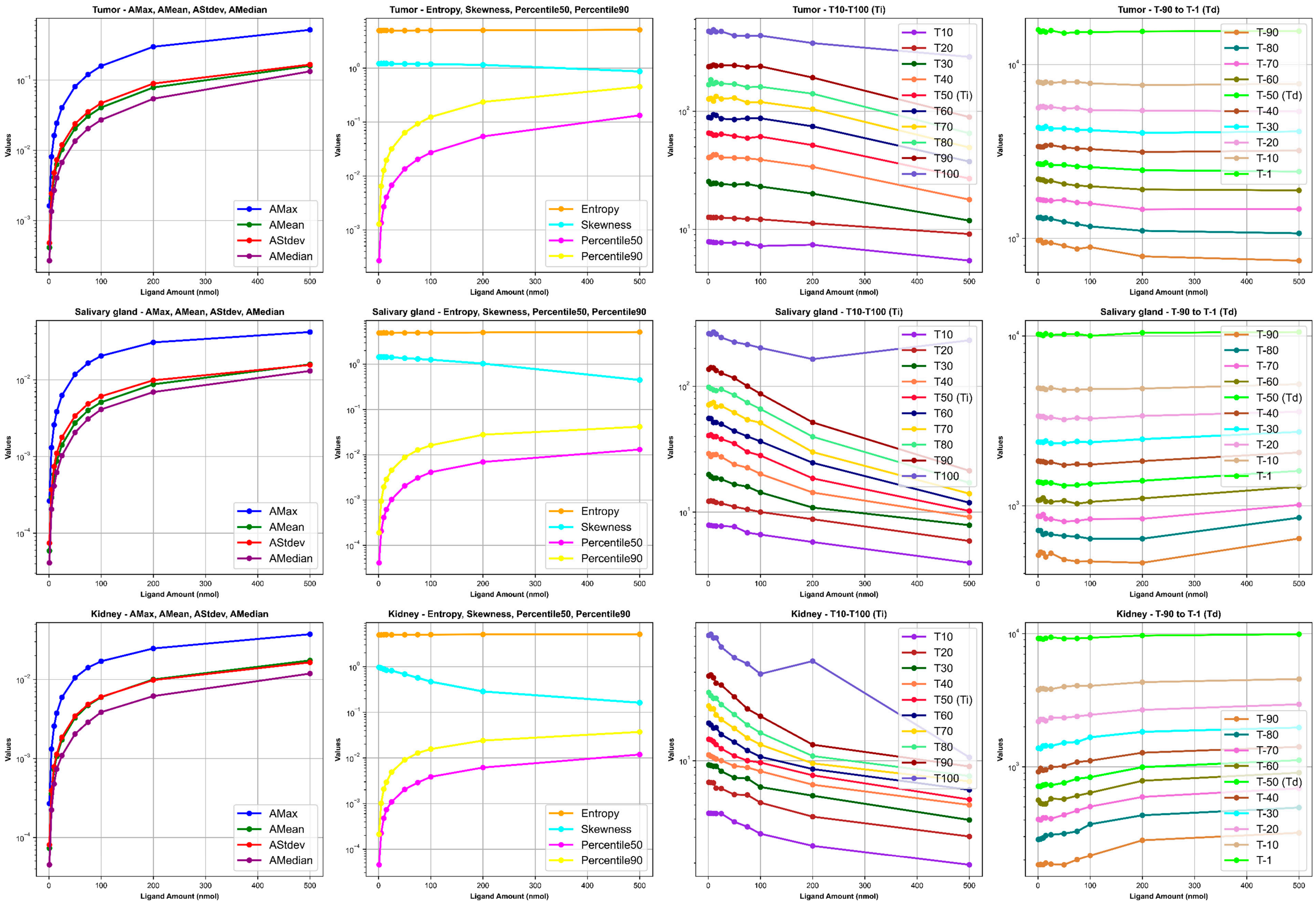

Figure S9. The impact of changes in Ligand Amount (nmol) on time activity curve (TAC) features. Ti and Td are increase and decrease half-time respectively.

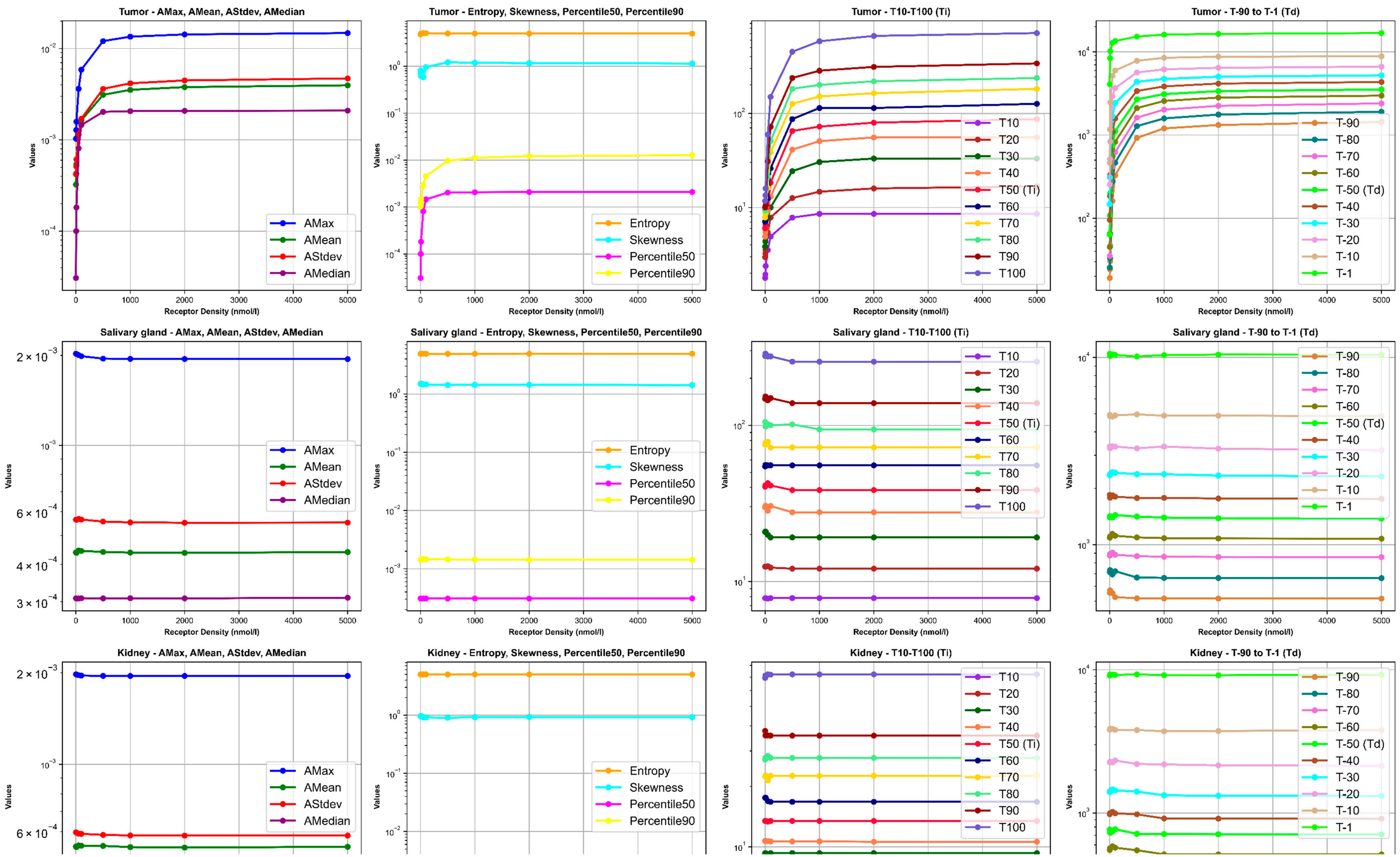

Figure S10. The impact of changes in Tumor Receptor Density (nmol/l) on time activity curve (TAC) features. Ti and Td are increase and decrease half-time respectively.

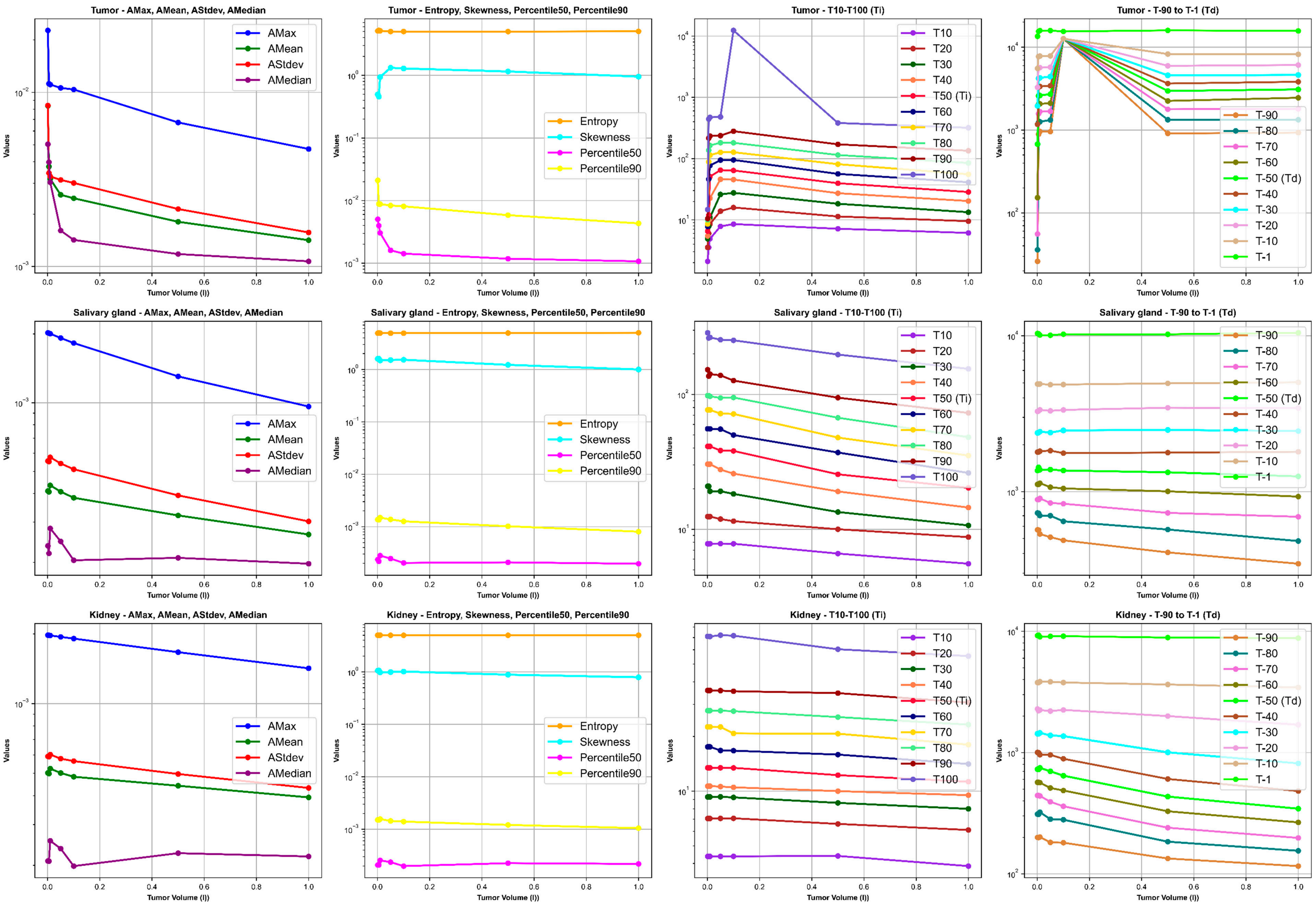

Figure S11. The impact of changes in *Tumor Volume (l)* on time activity curve (TAC) features. Ti and Td are increase and decrease half-time respectively.

References

1. Solanki JH, Tritt T, Pasternack JB, Kim JJ, Leung CN, Domogauer JD, et al. Cellular Response to Exponentially Increasing and Decreasing Dose Rates: Implications for Treatment Planning in Targeted Radionuclide Therapy. Radiat Res. 2017 Aug;188(2):221–34.
