# Supplementary material for: Model-Informed Radiopharmaceutical Therapy Optimization: A Study on the Impact of PBPK Model Parameters on Physical, Biological, and Statistical Measures in 177Lu-PSMA Therapy
Source: Cancers (Basel). 2024 Sep 10;16(18):3120. doi: 10.3390/cancers16183120 (PMC11430653; doi:10.3390/cancers16183120)
Supplement: Supplementary file 1 [file cancers-16-03120-s001.zip › Supplementary_Tables.pdf]

Supplementary Tables

Table S1. Model parameters for default tumor and normal organs.

Parameter	Tumors	Default Values for Normal Organs		
		Kidneys	Salivary glands	Unit
**Association rate**	0.046	Same as tumor	Same as tumor	l/nmol/min
**Internalization rate**	0.001	Same as tumor	Same as tumor	l/min
**Serum protein binding rate**	0.00047	Same as tumor	Same as tumor	1/min
**Release rate**	0.00024	0.00037	0.00037	1/min
**Ligand amount**	7.5	Same as tumor	Same as tumor	nmol
**Receptor density**	500	31	41	nmol/l

Table S2. Specific Values used in this study.

Organ	Volume (L)	S Value (Gy/min/MBq)	μ (1/min)	α/β (Gy)	Reference
**Tumor**	0.001	0.001332	0.0231	2.5	(1)
	0.005	0.000272			
	0.01	0.000137			
	0.035	3.99×10^{-5}			
	0.05	2.81×10^{-5}			
	0.1	1.42×10^{-5}			
	0.5	2.89×10^{-6}			
	1	1.46×10^{-6}			
**Salivary gland**	0.021	6.90×10^{-5}	0.0077	4.5	(1)
**Kidney**	0.3311	4.82×10^{-6}	0.004125	3.9	(1)

References

1. Kletting P, Schuchardt C, Kulkarni HR, Shahinfar M, Singh A, Glatting G, et al. Investigating the Effect of Ligand Amount and Injected Therapeutic Activity: A Simulation Study for 177Lu-Labeled PSMA-Targeting Peptides. PLoS One. 2016 Sep 9;11(9):e0162303.
